# Loss of the mitochondrial SAM transporter reveals a lipoylation-dependent metabolic vulnerability in the postnatal heart

**DOI:** 10.1126/sciadv.aeg8792

**Published:** 2026-08-26

**Authors:** Anastasia Rumyantseva, Trevor S. Tippetts, Alissa Wilhalm, William Carter, Marco Moedas, Florian A. Rosenberger, David Moore, Ákos Végvári, Yvonne Hinze, Linden Muellner-Wong, David Alsina, Rolf Wibom, Rainah Winston, Thomas P. Mathews, Lars H. Lund, Daniel C. Andersson, Gianluigi Pironti, Anna Wedell, Ralph J. DeBerardinis, Christoph Freyer, Anna Wredenberg

**Affiliations:** ^1^ Department of Medical Biochemistry and Biophysics, Karolinska Institutet, 171 76 Stockholm, Sweden.; ^2^ Children’s Medical Center Research Institute, University of Texas Southwestern Medical Center, Dallas, TX 75390, USA.; ^3^ Centre for Inherited Metabolic Diseases, Karolinska University Hospital, 171 76 Stockholm, Sweden.; ^4^ Max-Planck Institute for Biology of Ageing, 50931 Cologne, Germany.; ^5^ Department of Medicine, Karolinska Institutet, 171 65 Stockholm, Sweden.; ^6^ Cardiology Unit; Heart, Vascular, and Neuro Theme, Karolinska University Hospital, 171 76 Stockholm, Sweden.; ^7^ Department of Physiology and Pharmacology, Karolinska Institutet, 171 76 Stockholm, Sweden.; ^8^ Department of Molecular Medicine and Surgery, Karolinska Institutet, 171 76 Stockholm, Sweden.; ^9^ Howard Hughes Medical Institute, University of Texas Southwestern Medical Center, Dallas, TX 75390, USA.; ^10^ Eugene McDermott Center for Human Growth and Development, University of Texas, Southwestern Medical Center, Dallas, TX 75390, USA.

## Abstract

The neonatal heart experiences rapid metabolic growth after birth to meet increasing energetic and biosynthetic demands. How mitochondrial cofactor availability limits this transition remains unclear. Here, we demonstrate that mitochondrial *S*-adenosylmethionine (mitoSAM) import through SLC25A26 becomes limiting shortly after birth and specifically restricts protein lipoylation, although other mitoSAM-dependent processes are partially preserved. Loss of *Slc25a26* impaired lipoylation-dependent flux through pyruvate and α-ketoglutarate dehydrogenases, restricting tricarboxylic acid cycle carbon entry and depleting aspartate and nucleotide pools. Conversely, mitochondrial gene expression remained intact, and respiratory chain enzyme activities showed partial impairment, indicating that lipoylation is the most mitoSAM-sensitive pathway during postnatal heart adaptation. These metabolic limitations were linked to sustained cardiomyocyte cell–cycle activity, delayed structural maturation, and early cardiomyopathy. Supplementing with medium-chain triglycerides during the suckling-to-weaning transition partially stabilized metabolism and prolonged survival. Overall, our findings identify a stage-specific metabolic vulnerability in the postnatal heart characterized by hierarchical mitoSAM utilization within the mitochondria.

## INTRODUCTION

During the early postnatal period, the mammalian heart experiences a rapid metabolic shift to meet the increased energetic demands of continuous contraction. At birth, increased oxygen availability and a shift in nutrient supply, from placental glucose and lactate to lipid-rich milk and later carbohydrate-based solid foods, create new metabolic demands on the neonatal heart. To adapt, cardiomyocytes transition from mainly glycolytic metabolism in utero to mitochondrial oxidative metabolism after birth, marked by increased tricarboxylic acid (TCA) cycle activity and oxidative phosphorylation (OXPHOS) (*1*–*4*). This shift involves substantial mitochondrial remodeling, reflecting the need to expand metabolic capacity to support changing energetic and biosynthetic demands (*5*). As a result, effective growth and maturation of cardiomyocytes depend on increasing mitochondrial oxidative capacity, with the adult heart relying primarily on fatty acid oxidation and continuous TCA cycle activity to sustain contractile function (*6*, *7*).

Extensive changes in transcription and metabolism occur during this postnatal transition, and multiple studies have highlighted the importance of mitochondrial oxidative metabolism and TCA cycle flux for cardiac maturation (*8*–*10*). However, postnatal cardiomyocytes do not simply increase adenosine triphosphate (ATP) production to meet the growing contractile demands (*11*). Instead, they must support oxidative energy conversion while providing biosynthetic precursors required for cell growth and, temporarily, for continued proliferation during a brief postnatal period when cardiomyocytes retain their regenerative potential before fully maturing (*12*, *13*). How mitochondrial metabolism coordinates oxidative ATP production with TCA-linked building blocks during this stage of development remains unclear (*3*, *7*).

This question is especially relevant in the heart, which shows limited metabolic flexibility and a strong reliance on mitochondrial function (*14*–*16*). During early postnatal development, changes in nutrient supply and cofactor availability can therefore disproportionately affect cardiomyocyte growth and maturation (*17*–*22*). Supporting this idea, the TCA cycle intermediate α-ketoglutarate (αKG) has been associated with proliferative potential, including through αKG-dependent dioxygenases, demonstrating how mitochondrial metabolites link metabolic state to developmental processes (*23*). Similar principles also apply in the adult heart under pathological stress, where metabolic remodeling and changes in TCA cycle activity contribute to the progression of heart failure (*7*, *24*, *25*).

Together, these observations highlight a key unresolved problem in postnatal cardiac metabolism. While the shift from glycolysis to mitochondrial oxidative metabolism is well established, it remains unclear which upstream metabolic inputs and cofactor limitations determine whether cardiomyocytes successfully transition from hyperplastic growth to hypertrophic maturation in vivo.

*S*-adenosylmethionine (SAM) is a key metabolic cofactor synthesized from methionine and ATP that supports diverse methylation and radical-based reactions across cell compartments (*26*). In mitochondria, SAM (mitoSAM) has two distinct biochemical roles (*27*). First, it serves as a methyl donor for mitochondrial RNA and protein methyltransferases, thereby supporting mitochondrial gene expression, and for methylation reactions required during coenzyme Q biosynthesis. Second, mitoSAM is a substrate for radical SAM enzymes, most notably lipoic acid (LA) synthetase, which catalyzes mitochondrial lipoate biosynthesis. LA is a rare, highly conserved, posttranslational modification necessary for the activity of the glycine cleavage system H protein (GCSH) and the E2 subunits of several mitochondrial 2-oxoacid dehydrogenase complexes, including pyruvate dehydrogenase (PDHc), branched-chain α-ketoacid dehydrogenase (BCKDHc), 2-oxoadipate dehydrogenase (OADHc), and 2-oxoglutarate dehydrogenase (OGDHc), also known as αKG dehydrogenase (*28*). In these complexes, LA is covalently bound to the E2 subunits and serves as a flexible “swinging arm” that shuttles reaction intermediates between active sites, thereby enabling coordinated redox reactions and acyl-group transfer (*29*–*32*).

By supporting both mitochondrial methylation and lipoylation, mitoSAM couples gene expression to carbon flux through the TCA cycle. Although these processes are linked, they serve different metabolic needs. Proliferating cells may prioritize TCA-derived biosynthetic intermediates, while differentiated cells rely on sustained oxidative ATP production (*33*). How mitochondria allocate shared cofactors to balance these competing needs in vivo remains unknown. This raises the possibility that mitoSAM availability could serve as a metabolic decision point, enabling mitochondria to support specific developmental programs in response to metabolic context.

Here, we examined whether mitoSAM availability acts as a permissive factor in postnatal cardiac metabolic maturation. By using heart- and skeletal muscle-specific deletion of the mitoSAM transporter *Slc25a26*, we selectively restrict mitoSAM import during this developmental window. We demonstrate that reduced mitoSAM availability primarily impairs mitochondrial protein lipoylation, thereby limiting TCA cycle flux and related anabolic pathways essential for cardiomyocyte growth. Meanwhile, mitochondrial gene expression remains largely unaffected, and respiratory chain capacity is only partially reduced. These findings identify mitoSAM-dependent lipoylation as a critical metabolic gate that enables the postnatal heart to meet increasing oxidative demands and complete cardiomyocyte maturation.

## RESULTS

### Cardiomyocytes exhibit high *Slc25a26* expression and increased mitochondrial lipoylation during the postnatal metabolic transition

**Fig. 1.**
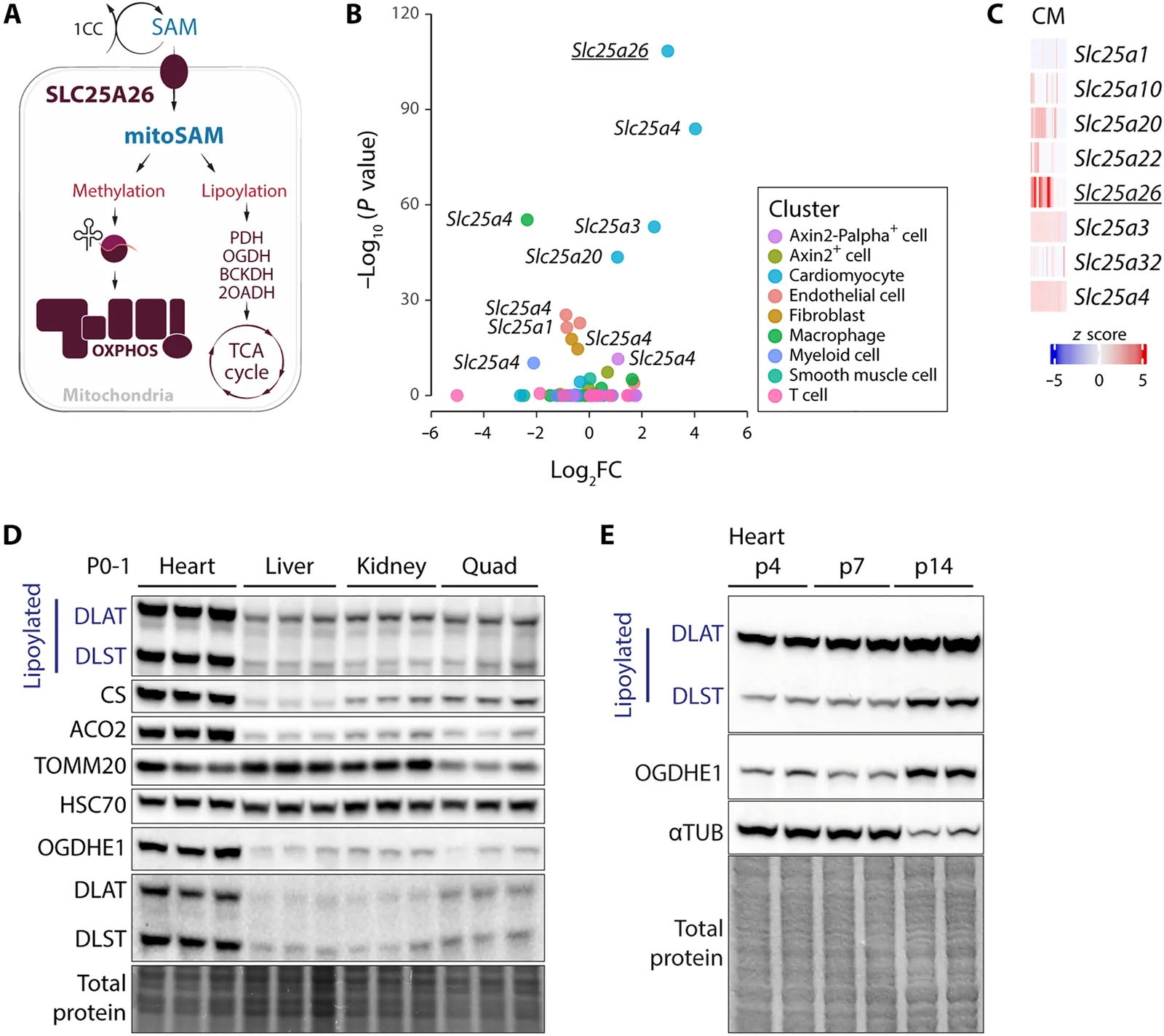
*Slc25a26* shows specific enrichment in the cardiomyocyte lineage. (**A**) Schematic diagram of SAM functions and its import into mitochondria. SAM is synthesized in the cytoplasm as part of the one-carbon cycle (1CC), imported via SLC25A26 into mitochondria, where it is used either as a methyl group donor or as a substrate for radical SAM enzymes, e.g., for the synthesis of LA; a cofactor of the PDHc, αKG dehydrogenase (OGDHc), branched-chain α-ketoacid dehydrogenase (BCKDHc), and 2-oxoadipate dehydrogenase (2-OADHc) complexes. (**B**) Volcano plot depicting log_2_FC for differential expression of selected SLC25 family members across clusters compared with the total population. Data were calculated from a publicly available dataset (*34*) using a Wilcoxon’s test with Bonferroni correction. (**C**) Heatmap showing expression levels of indicated SLC25 family members in cardiomyocyte (CM) lineage. (**D**) Immunoblot of control (Ctrl) tissue lysates from the heart, liver, kidney, and quadriceps (Quad) at P0-1 (*n* = 3) with indicated antibodies, DLAT, DLST, Citrate synthase (CS), mitochondrial aconitase (ACO2), translocase of outer mitochondrial membrane 20 (TOMM20) and αKG dehydrogenase E1 component (OGDHE1). HSC70 served as the loading control. (**E**) Immunoblot of Ctrl heart lysates at the indicated time points, probed with the indicated antibodies; α-tubulin (αTUB) was used as the loading control.

The postnatal heart rapidly increases mitochondrial mass and oxidative capacity to meet rising energetic and biosynthetic needs. Given its dual role within mitochondria (Fig. 1A), we hypothesized that mitoSAM availability might become limiting during this transition, leading us to predict that cardiomyocytes would show features indicative of greater reliance on mitoSAM-dependent processes. We therefore first asked whether the cardiomyocyte lineage displays molecular features consistent with increased mitoSAM demand.

Analysis of a publicly available single-cell RNA sequencing (RNA-seq) dataset covering 20 mouse tissues (*34*) revealed that *Slc25a26*, encoding the mitoSAM transporter, is among the most highly enriched SLC25 family members in cardiomyocytes (Fig. 1B and fig. S1A). To account for differences in mitochondrial content across tissues, we compared *Slc25a26* expression in cardiomyocytes with that of other mitochondrial transporters. Only the phosphate carrier (*Slc25a3*) and the adenosine diphosphate (ADP)/ATP translocase (*Slc25a4*) (Fig. 1C and fig. S1B) had similar expression levels, indicating a disproportionately high need for mitoSAM import relative to mitochondrial mass.

The enrichment of *Slc25a26* in cardiomyocytes likely reflects their adaptation to the postnatal shift toward oxidative metabolism. We therefore investigated whether mitoSAM levels themselves change during this transition. Although cellular SAM pools have been shown to fluctuate significantly during early postnatal development (*35*–*37*), targeted metabolomics revealed that mitoSAM levels remained remarkably stable from birth to weaning (fig. S1C). This stability suggests tight homeostatic control amid the rapid increase in mitochondrial mass and OXPHOS activity during this period.

Given the central role of the TCA cycle in energy metabolism, we assessed mitoSAM-dependent lipoylation, required for the activity of PDHc and OGDHc. Both lipoylation and the overall protein levels of their E2 subunits [dihydrolipoamide *S*-acetyltransferase (DLAT) and dihydrolipoamide *S*-succinyltransferase (DLST), respectively] were markedly higher in perinatal hearts than in other tissues (Fig. 1D) and increased further from postnatal day 4 (P4) to P14 (Fig. 1E), coinciding with the postnatal rise in oxidative metabolism (*3*, *10*). Consistent with this, the levels of additional TCA cycle enzymes, such as citrate synthase and mitochondrial aconitase (ACO2), were also increased in the heart (Fig. 1D). Collectively, these findings identify *Slc25a26* as highly enriched in the cardiomyocyte lineage, demonstrating that mitoSAM levels are consistently maintained despite postnatal remodeling, and show a coordinated increase in TCA cycle protein abundance and lipoylation during postnatal heart maturation.

### Loss of *Slc25a26* selectively impairs cardiac lipoylation before mitochondrial gene expression is affected

**Fig. 2.**
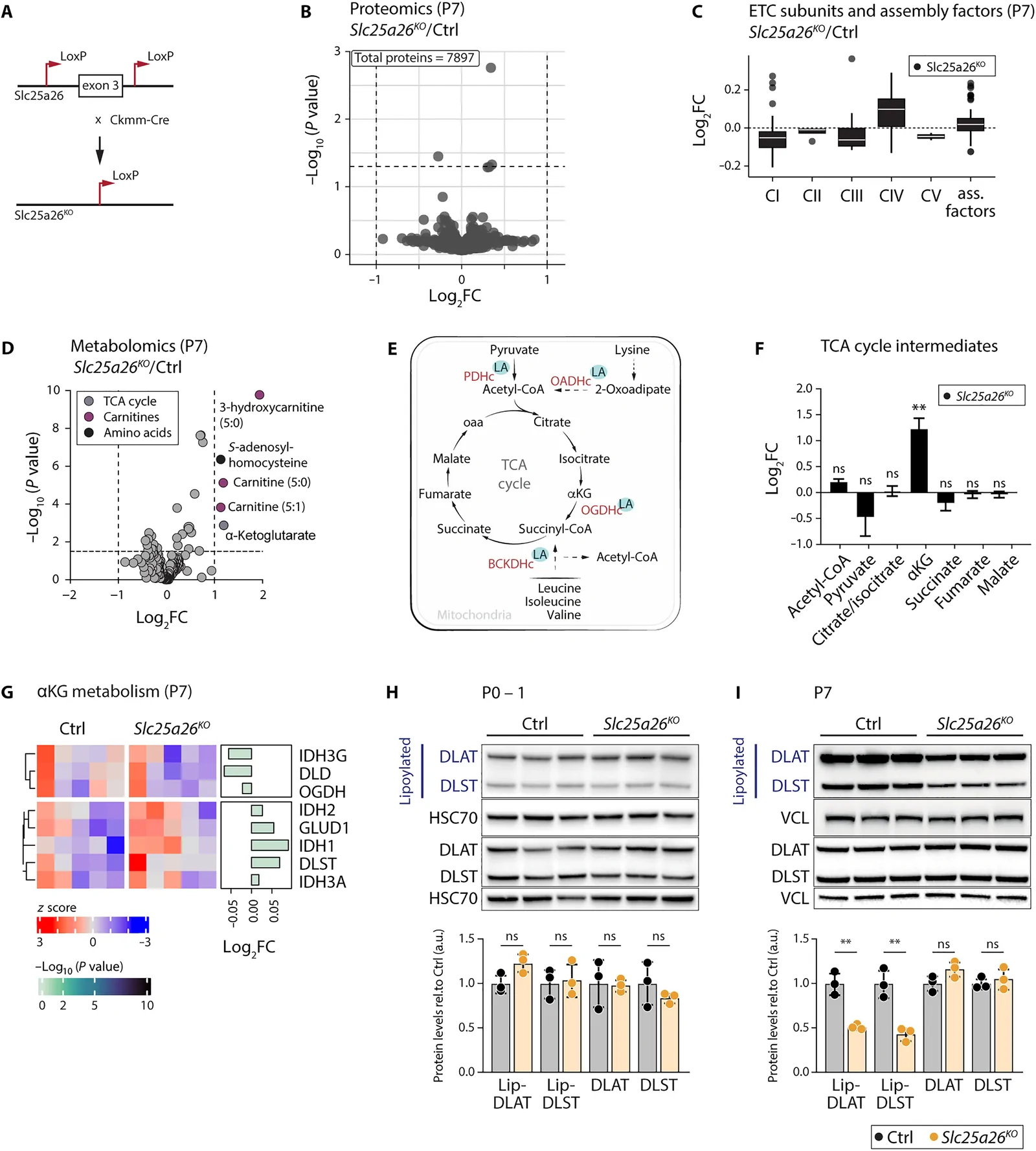
Lipoylation defect precedes impairment of mitochondrial gene expression in *Slc25a26^KO^* hearts. (**A**) Schematic diagram of breeding for *Slc25a26^KO^* mice. (**B**) LC-MS/MS label-free proteomics of whole heart, volcano plot comparing *Slc25a26^KO^* with Ctrl at P7 (*n* = 5 per genotype). (**C**) Boxplots of ETC subunits and assembly factors in *Slc25a26^KO^* heart proteome at P7, showing log_2_FC relative to Ctrl. (**D**) Volcano plot of untargeted metabolome data, displaying differentially abundant metabolites in *Slc25a26^KO^* versus Ctrl hearts at P7 (*n* = 6 per genotype). Metabolites are color-coded by category. (**E**) Schematic diagram of TCA cycle highlighting lipoylated (LA) enzymes. (**F**) Log_2_FC of TCA cycle intermediates in untargeted metabolomics in *Slc25a26^KO^* hearts at P7 relative to Ctrl. (**G**) Heatmap of relative *z* score for proteins involved in αKG metabolism from heart proteomics data. (**H** and **I**) Immunoblots of heart lysates from Ctrl and *Slc25a26^KO^* mice, showing lipoylated and apo-forms of DLAT and DLST at (H) P0-1 and (I) P7, with vinculin (VCL) or HSC70 as loading controls. Quantifications are shown below (*n* = 3 per genotype). Data in (E) are expressed as log_2_FC ± SEM and analyzed by two-tailed unpaired Student’s *t* test; ns, not significant, **P < 0.01. a.u., arbitrary units.

The high expression of *Slc25a26* in cardiomyocytes, along with the postnatal increase in protein lipoylation, suggests that mitoSAM transport may become limiting during postnatal remodeling. To examine the functional role of mitoSAM transport, we crossed *Slc25a26*^loxP/loxP^ mice (*38*) with transgenic animals expressing *Cre* recombinase driven by the *Ckmm-NLS* promoter from embryonic day 13.5 (*39*). The *Ckmm-NLS-Cre* line is widely used for cardiomyocyte-specific gene deletion and has not been linked to inherent cardiac developmental abnormalities in previous studies (*40*). Efficient recombination was observed in the heart and skeletal muscle but not in the liver (Fig. 2A and fig. S2A). Conditional knockout mice (hereafter *Slc25a26^KO^*) were born at expected Mendelian ratios and appeared indistinguishable from control littermates at P7, showing no signs of cardiomyopathy or growth delay (fig. S2, B to D).

We recently reported that mitoSAM-dependent methylation promotes mitochondrial ribosome assembly and translation in mouse embryonic fibroblasts and skeletal muscle (*41*). In contrast, label-free liquid chromatography-tandem mass spectrometry (LC-MS/MS) proteomics and immunoblot analysis in P7 *Slc25a26^KO^* hearts showed no major changes in electron transport chain (ETC) subunit abundance (Fig. 2, B and C; fig. S2E; and data S1). Mitochondrial transcript steady-state levels remained unchanged, except for a slight increase in mt-*Nd3* transcripts (fig. S2F). Moreover, mitochondrial stress markers (*Atf4*, *Atf5*, and *Chop*) were not induced (fig. S2G), and proteomic analysis revealed no notable differences in ETC accessory proteins, stress-response factors, or the cardiac contractile proteome (fig. S3, A and B). Overall, these findings suggest that loss of mitoSAM import does not impair mitochondrial gene expression or activate stress signaling in the early postnatal heart.

Given the absence of obvious pathology at P7, we next asked whether early metabolic changes occur before any structural or transcriptional defects. Untargeted metabolomics identified only five metabolites that were significantly altered in P7 *Slc25a26^KO^* hearts (Fig. 2D, fig. S3C, and data S2). Along with several C5-carnitines, reported to be byproducts of incomplete branched-chain amino acid oxidation (*42*), we observed a twofold increase of αKG. Other TCA cycle intermediates and 2-hydroxyglutarate (2-HG) remained unchanged, as did the protein levels of isocitrate dehydrogenase, glutamate dehydrogenase, and OGDHc subunits (Fig. 2, E to G), indicating a functional impairment rather than a change in OGDHc expression.

Since OGDHc activity depends on lipoylation of its E2 subunit (DLST), we next examined protein lipoylation during early postnatal development. Lipoylation was normal at birth but started to decline in *Slc25a26^KO^* hearts by P4 and continued to decrease throughout the first postnatal week (Fig. 2, H and I, and fig. S3D), coinciding with the shift toward oxidative metabolism (*3*, *10*). This occurred despite only a mild reduction in mitoSAM levels at P7 (fig. S3E), indicating that increasing metabolic demand reveals the vulnerability of the lipoylation pathway to reduced mitoSAM availability. Consistent with impaired SAM turnover, mitochondrial *S*-adenosylhomocysteine (SAH) levels were higher, aligning with the increased SAH observed through untargeted metabolomics (Fig. 2D and fig. S3E).

Therefore, cardiomyocyte-specific loss of *Slc25a26* leads to an early defect in protein lipoylation that precedes detectable changes in mitochondrial gene expression or ETC subunit abundance. These results highlight lipoylation as an early, mitoSAM-sensitive step in cardiac metabolism during the postnatal period.

### *Slc25a26* ablation causes early-onset cardiomyopathy in the postnatal heart

**Fig. 3.**
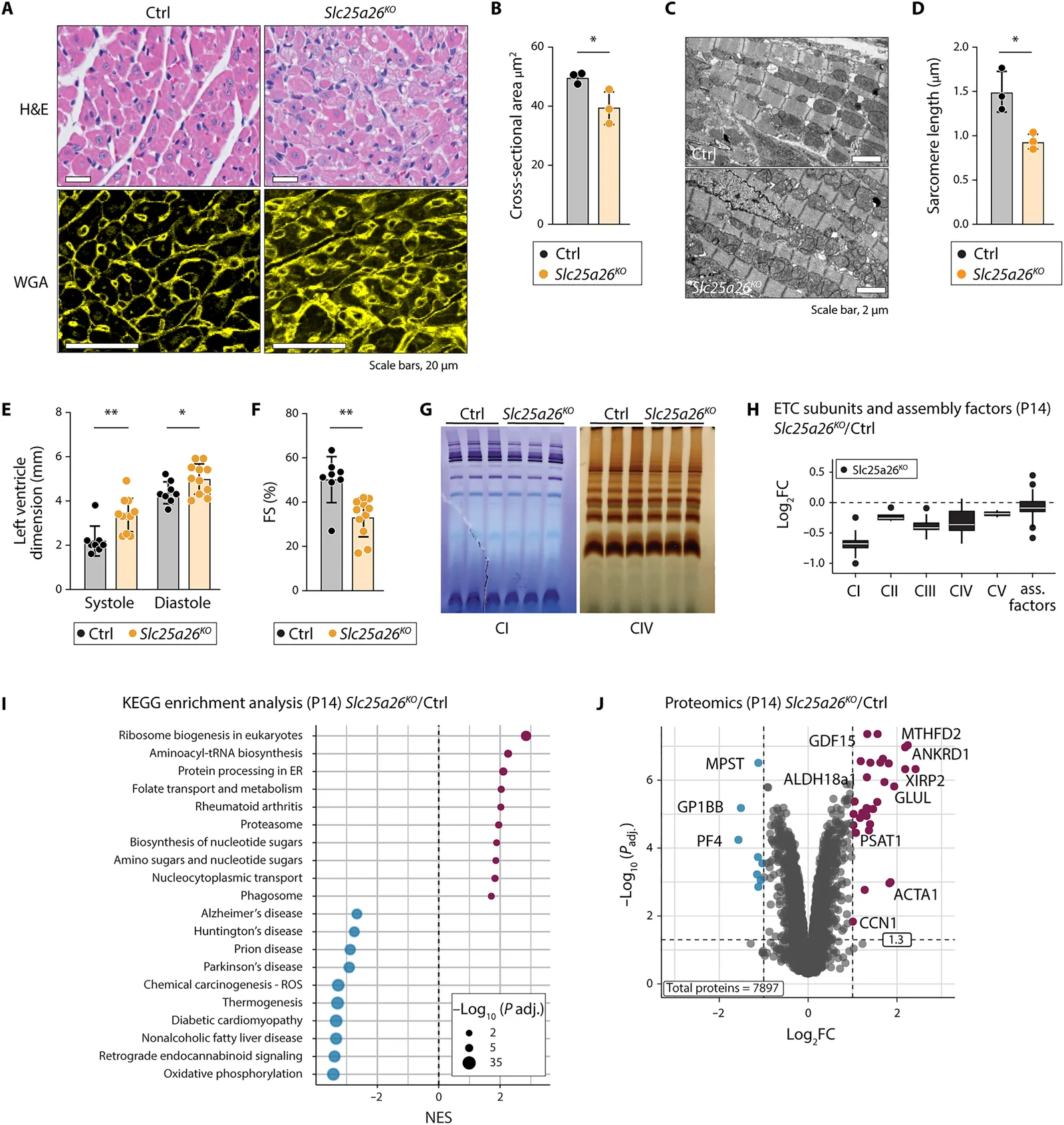
Loss of *Slc25a26* causes early lethality and cardiomyopathy. (**A**) Representative images of hematoxylin and eosin (H&E) and WGA staining in heart sections of Ctrl and *Slc25a26^KO^* mice at P14 (*n* = 3 per genotype; scale bars, 20 μm). (**B**) Quantification of cardiomyocyte cross-sectional area in Ctrl and *Slc25a26^KO^* mice (*n* = 3 per genotype). (**C**) Representative images of transmission electron micrographs of heart sections from Ctrl and *Slc25a26^KO^* mice at P14 (*n* = 3 per genotype; scale bars, 2 μm). (**D**) Quantification of sarcomere length in cardiomyocytes from Ctrl and *Slc25a26^KO^* mice (*n* = 3 per genotype). (**E**) Echocardiographic analysis of Ctrl (*n* = 8) and *Slc25a26^KO^* mice (*n* = 11) at P12, examining left ventricular (LV) dimensions during diastole and systole. (**F**) Fractional shortening of the LV in Ctrl (*n* = 8) and *Slc25a26^KO^* (*n* = 11) hearts at P12. (**G**) Enzymatic in-gel activity staining for CI and CIV in digitonin-solubilized cardiac mitochondria at P14, separated by BN-PAGE (*n* = 3 per genotype). (**H**) LC-MS/MS label-free whole-heart proteomics. Boxplots of ETC subunits and assembly factors in *Slc25a26^KO^* hearts relative to controls at P14 (*n* = 5 for Ctrl, *n* = 4 for *Slc25a26^KO^*). (**I**) KEGG pathway enrichment analysis of significantly up-regulated (purple) or down-regulated (blue) proteins in *Slc25a26^KO^* hearts. (**J**) Volcano plot of differentially expressed proteins in *Slc25a26^KO^* hearts compared with controls at P14, showing significantly changed proteins (log_2_FC > 1 and log_2_FC < −1, *P*_adj_ < 0.05) in purple (increased) and blue (decreased). Data in (B), (D), and (E) are means ± SD; each dot represents an individual mouse. Two-tailed unpaired Student’s *t* test; **P < 0.01, *P < 0.05.

The progressive loss of protein lipoylation in *Slc25a26^KO^* hearts prompted us to examine the physiological consequences during the postnatal period. Although *Slc25a26^KO^* mice appeared healthy at birth, they showed increased mortality shortly after P14. By P14, the heart weight–to–body weight ratio was significantly increased, due to increased cardiac mass (fig. S4, A to C). Histological analysis revealed vacuolar degeneration of cardiomyocytes, and wheat germ agglutinin (WGA) staining demonstrated a notable decrease in cardiomyocyte cross-sectional area (Fig. 3, A and B). In addition, ultrastructural analysis using transmission electron microscopy identified a significant reduction in sarcomere length in *Slc25a26^KO^* cardiomyocytes (Fig. 3, C and D). Echocardiography was performed at P12 to avoid complications related to anesthesia. *Slc25a26^KO^* hearts displayed enlarged left ventricular dimensions, decreased fractional shortening, and thinning of the interventricular septum, while posterior wall thickness remained unchanged (Fig. 3, E and F, and fig. S4D). Together, these results demonstrate the development of early-onset cardiomyopathy during the postnatal period.

Given the severity of the phenotype, we next assessed mitochondrial respiratory chain function. In-gel activity assays of complexes I and IV revealed preserved supercomplex assembly and only mild reductions in enzymatic activity in *Slc25a26^KO^* hearts (Fig. 3G). Spectrophotometric measurements confirmed moderate decreases in CI/CIII- and CII/CIII-linked activities (≈60% of control), likely reflecting reduced coenzyme Q availability (fig. S4, E and F), while complex IV activity was only modestly reduced (≈85% of control) (fig. S4E).

Consistent with these findings, steady-state levels of mitochondrial transcripts remained largely unchanged (fig. S4G), while proteomic analysis revealed a selective reduction in a subset of complex I subunits (log_2_FC ≈ −0.7), with other respiratory chain complexes being comparatively less affected (Fig. 3H, fig. S4H, and data S1). Kyoto Encyclopedia of Genes and Genomes (KEGG) pathway analysis of up-regulated proteins emphasized ribosome biogenesis, folate metabolism, and aminoacyl-tRNA biosynthesis (Fig. 3I). At the individual protein level, this was reflected by the induction of cardiac and mitochondrial stress markers (GDF15, ANKRD1, and MTHFD2) and enzymes involved in amino acid and one-carbon metabolism (PSAT1, GLUL, and ALDH18A1), indicating activation of stress-response pathways (Fig. 3J) (*43*).

Thus, *Slc25a26^KO^* hearts develop a rapidly progressive cardiomyopathy with early postnatal lethality. To identify the metabolic basis of this phenotype, we next examined mitochondrial and cellular metabolism at P14.

### *Slc25a26* ablation disrupts cardiac metabolism and lipoylation-dependent mitochondrial enzyme function

**Fig. 4.**
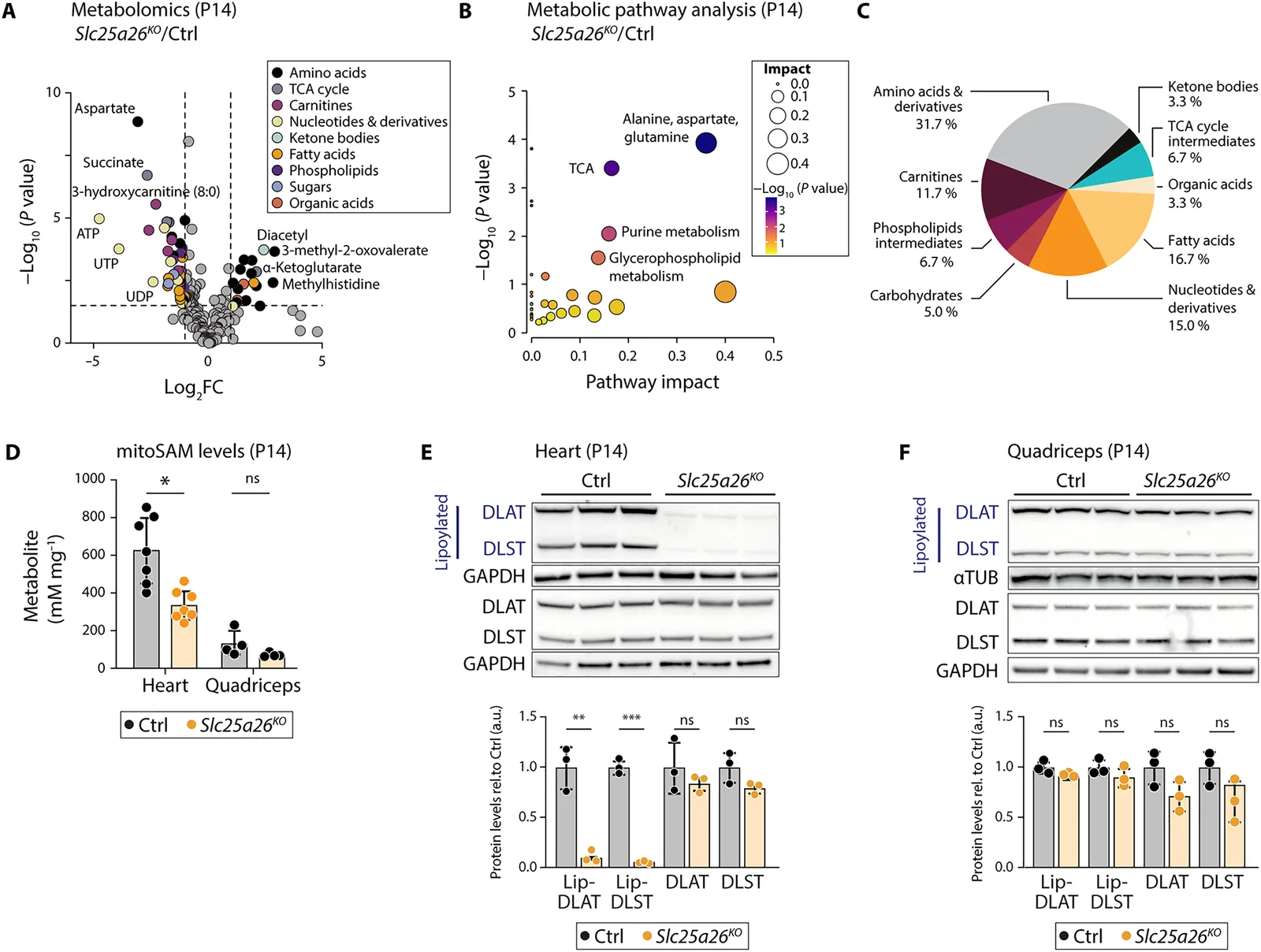
*Slc25a26* deficiency causes metabolic disturbance. (**A**) Volcano plot of the whole heart untargeted metabolome, showing metabolites with differential abundance in *Slc25a26^KO^* compared to control at P14. Significantly altered metabolites (log_2_FC >1 or < −1, *P* value < 0.05) are colored by categories (*n* = 7 for control, *n* = 5 for *Slc25a26^KO^*). (**B**) Metabolic pathway analysis of metabolites that differ in *Slc25a26^KO^* mice versus control. (**C**) Categories of significantly altered metabolites in *Slc25a26^KO^* hearts at P14. (**D**) Targeted metabolic analysis of mitoSAM levels in control and *Slc25a26^KO^* cardiac and quadriceps mitochondria (*n* = 7 per genotype for cardiac mitochondria, *n* = 4 per genotype for quadriceps mitochondria). (**E**) Immunoblot of control and *Slc25a26^KO^* heart lysates, showing lipoylated and apo-forms of DLAT and DLST at P14, with glyceraldehyde-3-phosphate dehydrogenase (GAPDH) as the loading control. Quantifications are shown below (*n* = 3 per genotype). (**F**) Immunoblot of control and *Slc25a26^KO^* skeletal muscle lysates, showing lipoylated and apo-forms of DLAT and DLST at P14, with αTUB and GAPDH as loading controls. Quantifications are shown below (*n* = 3 per genotype). Data in (D) are presented as means ± SD, with each dot representing an individual mouse, analyzed using a two-tailed unpaired Student’s *t* test; ns, not significant; *P < 0.05.

Untargeted metabolomics of P14 hearts revealed widespread metabolic alterations, with 51 of 221 detected metabolites significantly changed in *Slc25a26^KO^* hearts compared to controls (Fig. 4A, fig. S5A, and data S2). Accumulation of αKG and branched-chain ketoacids, combined with reduced levels of succinate, aspartate, and nucleotide pools, is consistent with impaired flux through lipoylation-dependent pathways involving 2-oxoacid dehydrogenases OGDHc, BCKDHc, and OADHc (Fig. 4, A and B). Additional changes in acylcarnitines, fatty acids, and phospholipid intermediates likely reflected secondary effects of restricted carbon flux through the TCA cycle (Fig. 4C).

Unexpectedly, *Slc25a26^KO^* hearts maintained more than 50% of mitoSAM levels at P14 (Fig. 4D), yet lipoylation of DLAT and DLST was almost completely abolished in *Slc25a26^KO^* hearts at P14 (Fig. 4E). In contrast, lipoylation remained intact in skeletal muscle from the same animals despite efficient *Slc25a26* deletion in this tissue (Fig. 4F and fig. S2A). Consistent with this, skeletal muscle presented with normal mitochondrial function, with steady-state levels of OXPHOS complex subunits and blue native polyacrylamide gel electrophoresis (BN-PAGE) analysis indicating normal assembly and in-gel activities of respiratory chain complexes (fig. S5, B and C). In addition, we previously reported that skeletal muscle-specific deletion of *Slc25a26* causes a slowly progressive myopathy with an approximate lifespan of three months, unlike the acute postnatal lethality observed here (*41*). These findings argue against a systemic metabolic contribution and instead point to a heart-specific metabolic vulnerability.

Collectively, these data identify lipoylation-dependent dehydrogenases as key metabolic bottlenecks in the heart following *Slc25a26* loss. Notably, this defect occurs despite retaining more than 50% of mitoSAM levels, demonstrating that lipoylation-dependent mitochondrial processes are highly sensitive to even partial reductions in mitoSAM availability during postnatal cardiac maturation (*44*–*46*).

### Loss of lipoylation alters local cysteine redox states in 2-oxoacid dehydrogenase complexes

Several antioxidant metabolites were decreased in *Slc25a26^KO^* hearts, indicating altered redox balance (fig. S5D). To assess whether this was related to changes in protein redox state, we performed redox proteomics to quantify cysteine oxidation (*46*). This analysis revealed a limited, highly specific pattern of redox changes, with only nine cysteine residues in mitochondrial proteins showing increased oxidation in *Slc25a26^KO^* hearts (fig. S5E and data S3). Notably, we identified all known lipoate-modified peptides, except for the GCSH protein. The oxidized cysteines were located near the conserved lipoylated lysines of OGDHc and PDHc subunits (fig. S5F), suggesting that lipoate modification may help stabilize local protein structure and protect adjacent cysteines from oxidation. Similar protective effects of lipoylation have been proposed in ex vivo studies (*44*, *45*).

In addition, we observed opposing redox shifts in malate dehydrogenase 2, which became more oxidized, and glutamate-oxaloacetate transaminase 2, which became more reduced. These are two key components of the malate-aspartate shuttle necessary for cardiac aspartate synthesis (*47*). These specific redox changes imply that lipoylation-dependent TCA cycle restriction indirectly interferes with mitochondrial-cytosolic redox coupling rather than causing widespread protein oxidation.

### *Slc25a26* deficiency disrupts nucleotide metabolism and prolongs cardiomyocyte cell–cycle activity

**Fig. 5.**
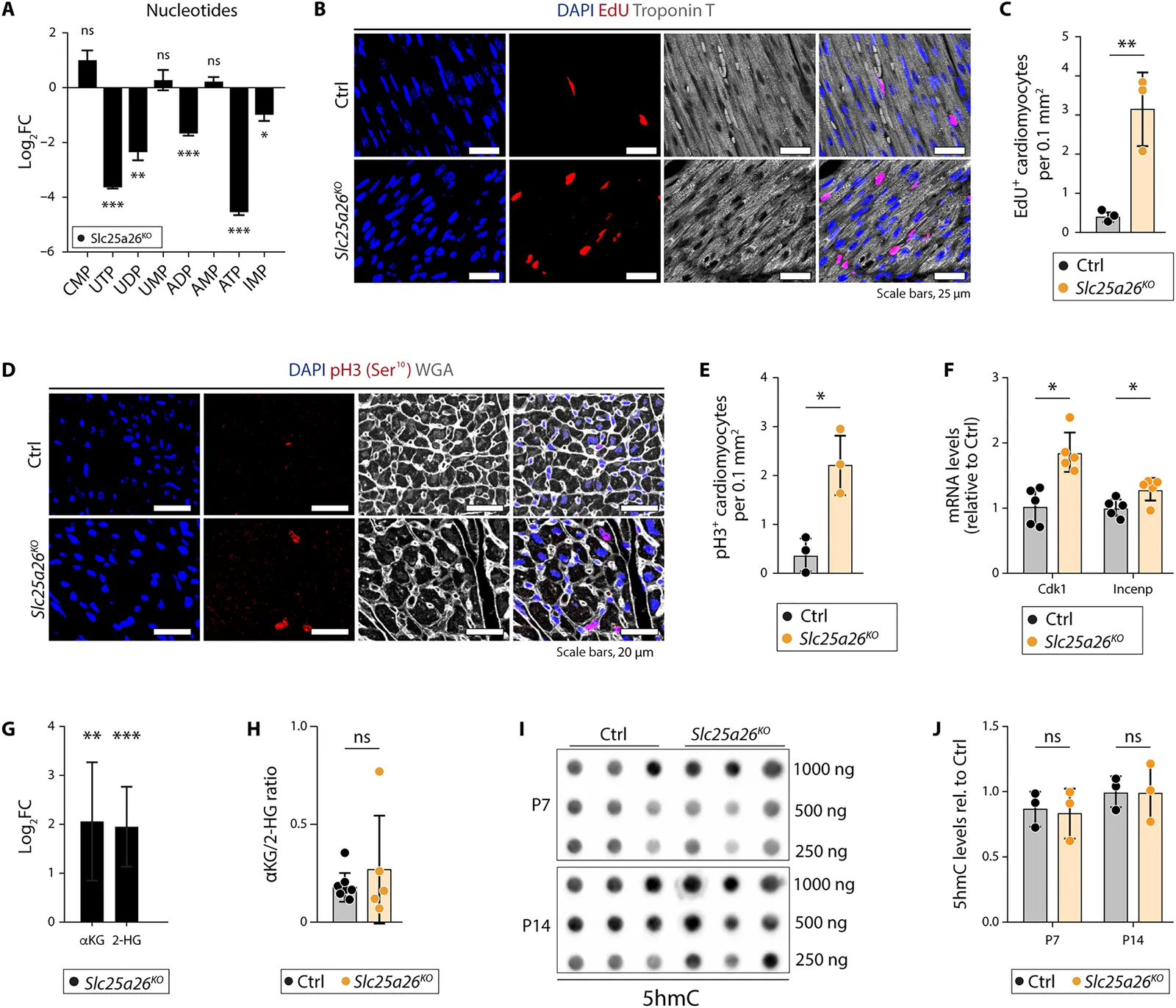
*Slc25a26* deficiency disrupts nucleotide metabolism and extends cardiomyocyte cell-cycle activity. (**A**) Log_2_FC of nucleotides in the untargeted metabolome of *Slc25a26^KO^* hearts compared with controls at P14. (**B**) Representative immunofluorescent images of P14 heart sections displaying EdU incorporation (EdU), cardiomyocytes (anti-cardiac troponin T), and nuclei [4′,6-diamidino-2-phenylindole (DAPI)]; scale bar, 25 μm. (**C**) Quantification of (B) EdU^+^ cardiomyocytes. (**D**) Representative images showing phosphorylated (Ser10) histone 3 (pH3), WGA, and DAPI (nuclei) staining of heart sections from control and *Slc25a26^KO^* mice at P14 (*n* = 3 per genotype; scale bar, 20 μm). (**E**) Quantification of pH3^+^ cardiomyocytes (*n* = 3 per genotype). (**F**) Transcript levels of specified genes in control and *Slc25a26^KO^* hearts at P14, analyzed using quantitative reverse transcription PCR (*n* = 5 per genotype). (**G**) Log_2_FC of αKG and 2-HG in the untargeted metabolome of *Slc25a26^KO^* hearts compared with controls at P14 (*n* = 7 for controls, *n* = 5 for *Slc25a26^KO^*). (**H**) Ratio of αKG to 2-HG in control and *Slc25a26^KO^* hearts at P14. (**I**) DNA dot blot analysis of 5hmC levels in control and *Slc25a26^KO^* hearts at P7 and P14. (**J**) A quantification of (I), normalized to the amount of DNA (*n* = 3 per genotype and time point). Data in (A), (G), and (H) are displayed as log_2_FC ± SEM, analyzed using two-tailed unpaired Student’s *t* test; ns, not significant, *P < 0.05, **P < 0.01, ***P < 0.001. Data in (C), (E), (F), and (I) represent mean ± SD, with each dot indicating an individual mouse, analyzed using two-tailed unpaired Student’s *t* test; *P < 0.05, **P < 0.01.

Given the central role of lipoylated dehydrogenases in maintaining TCA cycle flux, we next examined the downstream metabolic effects of *Slc25a26* loss. Nucleotide profiling showed significant depletion of ATP, uridine triphosphate (UTP), and uridine diphosphate (UDP), along with reduced levels of aspartate, a crucial substrate for nucleotide synthesis, and purine intermediates such as inosine monophosphate (IMP) and succinyladenosine (Figs. 4A and 5A and figs. S6A and S6B). Although adenine pools are frequently reduced in heart failure (*48*, *49*), *Slc25a26^KO^* hearts exhibited reductions not only in nucleotide precursors but also in degradation products such as adenosine and xanthine (fig. S6, A and B). Proteomic analysis further identified notable changes in nucleotide metabolism, including increased levels of enzymes involved in de novo purine and pyrimidine synthesis (IMPDH1, IMPDH2, TYMS, and CTPS1) and nucleotide sugar biosynthetic pathways (Fig. 3I and fig. S6, C and D), indicating a compensatory response to nucleotide depletion.

De novo nucleotide biosynthesis is energy intensive, and cardiomyocytes typically exit the cell cycle shortly after birth, relying mainly on nucleotide salvage pathways beyond P7 (*10*, *50*). We therefore hypothesized that the observed metabolic profile reflected a prolonged period of cardiomyocyte cell–cycle activity. Consistent with this, in vivo 5-ethynyl-2′-deoxyuridine (EdU) incorporation demonstrated that while control hearts showed very low levels of EdU incorporation by P14, *Slc25a26^KO^* hearts retained a significantly higher proportion of EdU-positive cardiomyocytes (Fig. 5, B and C). In addition, levels of phosphorylated histone H3 (pH3 Ser10) were elevated in *Slc25a26^KO^* hearts at P14, indicating increased entry into mitosis (Fig. 5, D and E). Furthermore, *Slc25a26^KO^* hearts exhibited significantly higher transcript levels of *Cdk1* and *Incenp*, suggesting ongoing cell-cycle progression (Fig. 5F). Together, these findings indicate that cardiomyocytes in *Slc25a26^KO^* hearts retain cell-cycle activity beyond the normal postnatal window of cell-cycle withdrawal.

In parallel with sustained cell-cycle activity, transcriptional and proteomic markers of cardiomyocyte maturation were altered. Transcript levels of adult sarcomeric isoforms and key calcium-handling genes (*Atp2a2*, *Ryr2*, and *Kcnip2*) were decreased, while fetal isoforms and stress-associated markers (*Nppa* and *Nppb*) were increased (fig. S7, A and B). Proteomic analysis confirmed down-regulation of proteins involved in cardiac muscle contraction (fig. S7C).

αKG has been shown to promote cardiomyocyte proliferation through αKG-dependent dioxygenases in neonatal and postinjury contexts (*19*, *23*). In *Slc25a26^KO^* hearts, αKG was already elevated at P7 and increased to approximately fourfold by P14 (Figs. 2F and 5G). 2-HG levels, a competitive inhibitor of αKG-dependent dioxygenases (*51*), were unchanged at P7 but increased by P14, resulting in no substantial change in the αKG/2-HG ratio at this stage (Fig. 5, G and H). Consistent with this, global 5-hydroxymethylcytosine (5hmC) levels remained unchanged at both P7 and P14 (Fig. 5, I and J).

Together, these data suggest that *Slc25a26* ablation causes a metabolome-first collapse driven by the loss of lipoylation-dependent TCA cycle flux, resulting in depletion of nucleotide pools while cardiomyocyte cell–cycle activity remains sustained. Although localized epigenetic effects cannot be ruled out, our semiquantitative analysis of global 5hmC abundance did not detect consistent differences between genotypes, supporting the view that the major phenotype is associated with metabolic dysfunction.

### MitoSAM-dependent lipoylation defines a substrate bottleneck that can be partially bypassed by MCT supplementation

Mice undergo a critical suckling-to-weaning transition around P12, during which milk rich in medium-chain triglycerides (MCTs) and long-chain triglycerides is gradually replaced by a carbohydrate-based chow diet (*52*). This transition coincides with rising cardiac energy demands, creating a dual metabolic challenge characterized by declining triglyceride availability and increased reliance on pyruvate- and glutamate-derived carbon entry into the TCA cycle (*24*, *53*). *Slc25a26^KO^* hearts are likely unable to accommodate this shift because of impaired activity of lipoylation-dependent dehydrogenase complexes (fig. S7D).

**Fig. 6.**
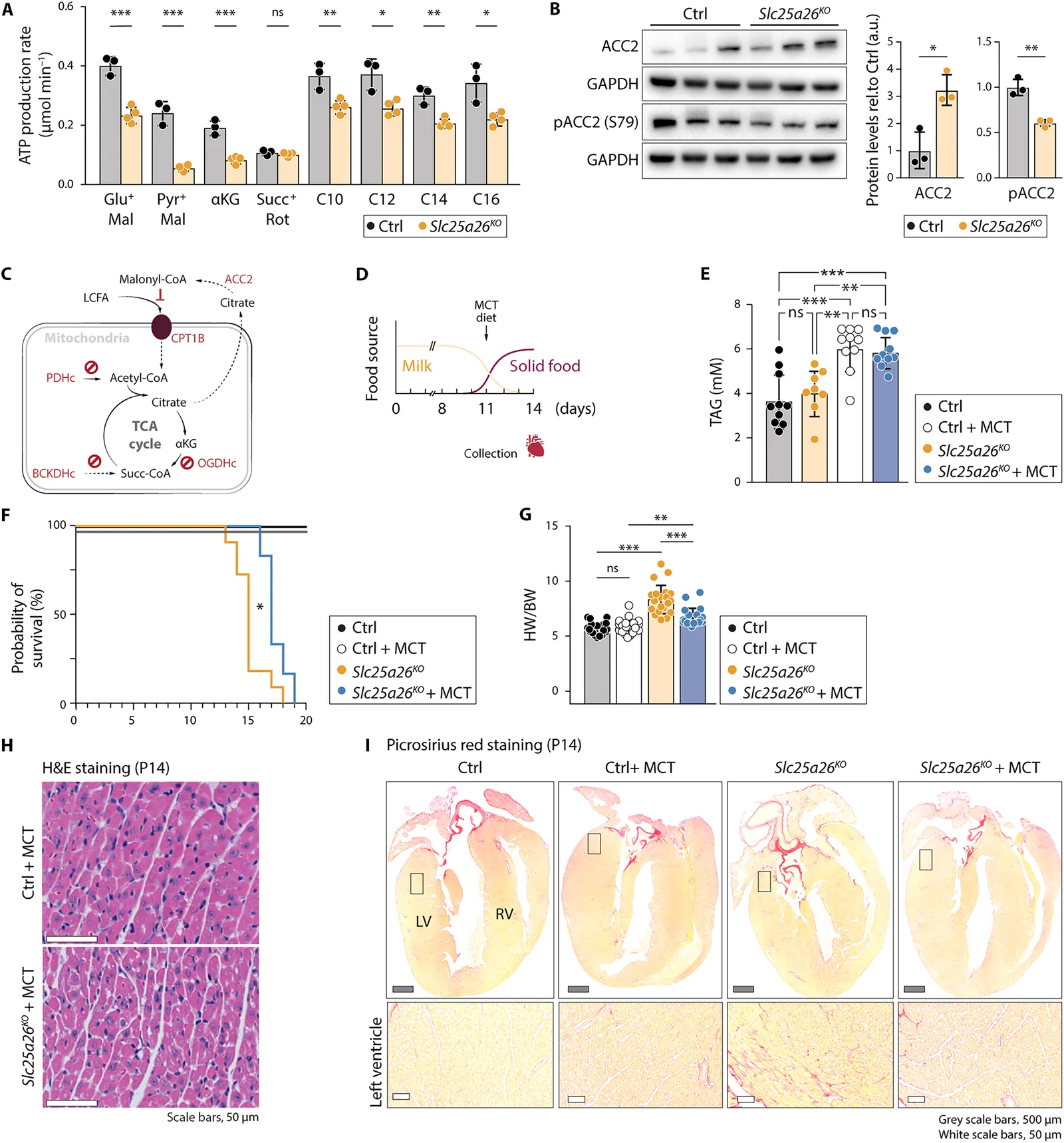
Dietary MCT supplementation improves survival and cardiac pathology in *Slc25a26^KO^* mice. (**A**) Ex vivo ATP production assay in Ctrl (*n* = 3) and *Slc25a26^KO^* (*n* = 4) P14 mitochondria supplied with indicated substrates. (**B**) Immunoblot of Ctrl and *Slc25a26^KO^* heart lysates, showing total and phosphorylated (S79) ACC2. GAPDH served as a loading control. Quantifications are shown on the right (*n* = 3 per genotype). (**C**) Scheme illustrating impaired cardiac substrate utilization in *Slc25a26^KO^* mice at P14. (**D**) MCT diet strategy. (**E**) Blood triglyceride levels in Ctrl and *Slc25a26^KO^* animals fed normal or MCT-enriched chow at P14 (*n* = 10, 10, 8, and 10 for Ctrl, Ctrl + MCT, *Slc25a26^KO^*, and *Slc25a26^KO^* + MCT, respectively). (**F**) Kaplan-Meier survival curve of *Slc25a26^KO^* mice (*n* = 11 for *Slc25a26^KO^* and *n* = 6 for *Slc25a26^KO^* + MCT), fed either normal or MCT-enriched chow, with Mantel-Cox log-rank test *P < 0.05. (**G**) Heart weight (HW) normalized to body weight (BW) of P14 mice with MCT diet (*n* = 21, 26, 20, and 18 for Ctrl, Ctrl + MCT, *Slc25a26^KO^*, and *Slc25a26^KO^* + MCT, respectively). (**H**) H&E staining of heart sections from *Slc25a26^KO^* and *Slc25a26^KO^* + MCT mice at P14. (**I**) Picrosirius red staining of hearts from control and *Slc25a26^KO^* and *Slc25a26^KO^* + MCT mice at P14 (*n* = 3 per genotype and treatment). Data in (A) are presented as means ± SD, with each dot representing an individual mouse, analyzed using a two-tailed unpaired Student’s *t* test; ns, not significant, *P < 0.05, **P < 0.01, ***P < 0.001. Data [(E) and (G)] are presented as means ± SD, with each dot representing an individual mouse, analyzed using a two-way analysis of variance (ANOVA) with Tukey’s multiple comparisons test; ns, not significant, *P < 0.05, **P < 0.01, ***P < 0.001.

To directly define this metabolic bottleneck, we measured substrate-specific ATP production in isolated cardiac mitochondria. ATP generation from succinate remained unchanged, while production from medium- or long-chain acylcarnitines was modestly reduced in *Slc25a26^KO^* mitochondria, indicating that *Slc25a26^KO^* hearts are unable to establish a fully mature cardiac metabolism. ATP production from substrates that rely on lipoylated enzymes such as PDHc (pyruvate + malate) or OGDHc (αKG + glutamate) were severely impaired (Fig. 6A), highlighting their rate-limiting role. Therefore, although the respiratory chain itself was largely functional, the loss of flux through lipoylation-dependent dehydrogenases prevented *Slc25a26^KO^* hearts from adapting to changing carbon source utilization during a stage when metabolic flexibility is essential.

In addition to impaired entry of pyruvate- and glutamate-derived carbon, *Slc25a26^KO^* hearts exhibited reduced pACC2 levels, consistent with increased ACC2 activity and potentially elevated malonyl–coenzyme A (CoA) production (Fig. 6B). Since malonyl-CoA inhibits the carnitine palmitoyltransferase CPT1B, this shift may further limit long-chain fatty acid oxidation (*54*, *55*). In agreement, high-resolution respirometry measurements in permeabilized left ventricular myocardium supplied with palmitoylcarnitine were reduced in *Slc25a26^KO^* hearts, confirming impaired oxidation of the long-chain fatty acid–derived substrates (fig. S7E). Along with the block at PDHc and OGDHc, these changes are likely to further limit substrate flexibility in the postnatal heart (Fig. 6C).

MCTs enter mitochondria independently of CPT1 (*8*, *56*). We therefore hypothesized that dietary supplementation with MCTs during the suckling-to-weaning transition could bypass impaired substrate entry into the TCA cycle and alleviate the emerging energetic crisis in *Slc25a26^KO^* hearts. To selectively supplement pups during the suckling-to-weaning transition, MCT-enriched pellets were placed in petri dishes on the cage floor from P11, while standard chow remained accessible only to the dam (Fig. 6D). Circulating triglyceride levels increased in both control and *Slc25a26^KO^* mice fed the MCT-enriched diet, consistent with effective ingestion and systemic availability of the supplemented lipids (Fig. 6E). MCT supplementation extended the survival of *Slc25a26^KO^* mice by approximately 15% (Fig. 6F) and significantly improved the heart weight–to–body weight ratio compared with chow-fed KO animals (Fig. 6G and fig. S8, A and B). Furthermore, MCT-fed *Slc25a26^KO^* mice exhibited reduced cardiomyocyte vacuolization and a morphology similar to the control hearts (Fig. 6H). Picrosirius red staining showed increased fibrosis localized to the left ventricle in the *Slc25a26^KO^* hearts, which was absent in MCT-treated *Slc25a26^KO^* hearts (Fig. 6I).

**Fig. 7.**
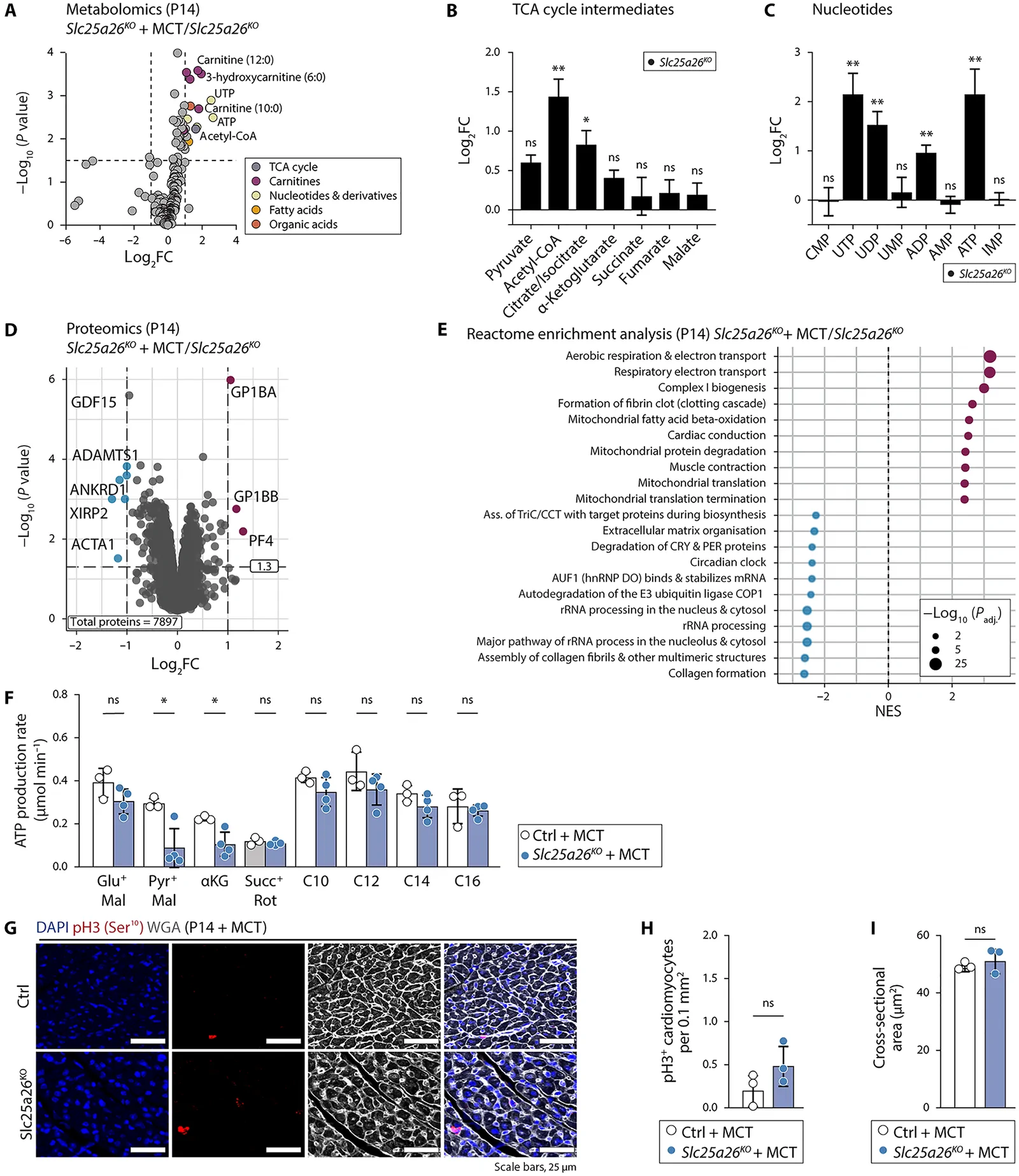
MCT-enriched food restores metabolomic and proteomic profiles and cardiomyocyte size. (**A**) Volcano plot of untargeted metabolome data, showing differentially abundant metabolites in the hearts of P14 *Slc25a26^KO^* mice fed on MCT-enriched versus normal chow. Significantly altered metabolites (log_2_FC >1 and log_2_FC < −1, *P* value < 0.05) are colored according to categories (*n* = 5 for *Slc25a26^KO^*, *n* = 6 for *Slc25a26^KO^* + MCT). (**B**) Log_2_FC of TCA cycle intermediates detected in the untargeted heart metabolome of P14 *Slc25a26^KO^* mice fed on MCT-enriched versus normal chow. (**C**) Log_2_FC of nucleotides detected in the untargeted heart metabolome of P14 *Slc25a26^KO^* mice fed on MCT-enriched versus normal chow. (**D**) LC-MS/MS label-free whole heart proteomic analysis. Volcano plot of differentially expressed proteins in hearts from P14 *Slc25a26^KO^* mice fed on MCT-enriched versus normal chow. Significantly changed proteins (log_2_FC >1 and log_2_FC < −1, *P*_adj_ < 0.05) are shown in purple (increased) and blue (decreased). (**E**) Reactome pathway enrichment analysis of significantly up- (purple) or down-regulated (blue) proteins in *Slc25a26^KO^* + MCT hearts. (**F**) Ex vivo ATP production assay in MCT-fed Ctrl (*n* = 3) and *Slc25a26^KO^* (*n* = 4) P14 mitochondria supplied with indicated substrates. (**G**) Representative images of pH3 (Ser^10^), WGA, and DAPI (nuclei) staining of heart sections from Ctrl and *Slc25a26^KO^* mice on MCT-enriched diet at P14 (*n* = 3 per genotype; scale bars, 25 μm). (**H**) Quantification of pH3^+^ cardiomyocytes (*n* = 3 per genotype). (**I**) Quantification of the cross-sectional area of cardiomyocytes in Ctrl and *Slc25a26^KO^* mice on MCT-enriched diet (*n* = 3 per genotype). Data in (B) and (C) are presented as log_2_FC ± SEM, two-tailed unpaired Student’s *t* test; ns, not significant, *P < 0.05, **P < 0.01. Data in (F), (H), and (I) are means ± SD, with each dot representing a single mouse, analyzed by two-tailed unpaired Student’s *t* test; ns, not significant.

At the metabolic level, MCT supplementation partially restored high-energy metabolites in *Slc25a26^KO^* hearts at P14, including ATP, UTP, and UDP (Fig. 7, A to C). Citrate levels were increased, consistent with enhanced acetyl-CoA availability, while downstream TCA cycle intermediates beyond αKG remained largely unchanged (Fig. 7B), indicating partial stabilization of proximal carbon entry rather than full restoration of TCA cycle flux. Consistent with improved metabolic capacity, levels of lipoylated DLAT and DLST were modestly but reproducibly increased in MCT-fed *Slc25a26^KO^* hearts compared with untreated controls (fig. S9A), suggesting that increased availability of medium-chain substrates may enhance residual lipoylation-dependent enzyme activity.

Proteomic analysis supported a partial restoration of cardiac structure and function, indicating decreased levels of sarcomeric stress and cardiomyopathy markers, and increased activity in pathways related to cardiac muscle contraction (Fig. 7, D and E; and fig. S9, B to D; and data S1). Consistent with the metabolic rescue, ATP production in isolated cardiac mitochondria from MCT-fed *Slc25a26^KO^* hearts was restored to control levels when supported by glutamate + malate and by medium- or long-chain acylcarnitines (Fig. 7F). In contrast, ATP production from substrates that depend directly on lipoylated dehydrogenases, including pyruvate + malate (PDHc) and αKG + glutamate (OGDHc), remained markedly impaired. These findings suggest that MCT supplementation primarily improves energy production by enhancing fatty acid–derived carbon flux rather than restoring lipoylation-dependent metabolism (Fig. 7F). Furthermore, phospho-histone H3 staining revealed that cardiomyocyte cell–cycle activity in MCT-fed *Slc25a26^KO^* hearts was comparable to the MCT-fed controls at P14, consistent with restoration of normal postnatal cell-cycle withdrawal (Fig. 7, G and H). Similarly, WGA staining showed that cardiomyocyte cross-sectional area was restored to control levels in MCT-fed *Slc25a26^KO^* hearts (Fig. 7I). Together, these findings demonstrate that dietary MCT supplementation partially restores metabolic homeostasis, promotes postnatal maturation of cardiomyocytes, and improves cardiac structure and survival in *Slc25a26*-deficient mice.

## DISCUSSION

The shift toward oxidative metabolism in the early postnatal heart requires coordinated transcriptional and metabolic reprogramming to meet rising energetic and biosynthetic needs. Previous studies have linked SAM availability to the regulation of chromatin accessibility and epigenetic remodeling during this transition (*35*–*37*). Our work extends these observations by identifying mitoSAM availability, rather than total cellular SAM levels, as a limiting cofactor in postnatal cardiac metabolic maturation, acting primarily by sustaining protein lipoylation.

MitoSAM sustains mitochondrial function through two distinct biochemical processes: mitochondrial methylation reactions and radical SAM-dependent LA biosynthesis. We show that even a modest reduction in mitoSAM levels selectively impairs protein lipoylation, creating a bottleneck at lipoylation-dependent 2-oxoacid dehydrogenases. This defect persists despite maintaining more than half of mitoSAM levels and occurs before widespread proteomic changes or activation of canonical mitochondrial stress responses, emphasizing lipoylation as a particularly vulnerable metabolic point during early postnatal life. Although *Slc25a26* was deleted during late embryogenesis, its functional consequences became apparent only after birth, coinciding with the heart’s increased dependence on oxidative metabolism. Furthermore, lipoylation is normal at birth and declines progressively only after P4, arguing against a primary developmental defect. Thus, our findings identify LA biosynthesis as a stage-specific metabolic vulnerability exposed during postnatal cardiac remodeling.

The selective vulnerability of lipoylation in the postnatal heart contrasts with our previous findings in skeletal muscle, where muscle-specific *Slc25a26* deletion primarily caused a mitochondrial defect due to impaired mitochondrial gene expression rather than lipoylation (*41*). Collectively, these findings suggest that distinct mitoSAM-dependent processes can become rate limiting, depending on tissue, context, metabolic demand, and developmental stage.

Biallelic variants in *SLC25A26* in pediatric and adult patients are associated with lactic acidosis, episodes of acute cardiopulmonary failure, progressive muscle weakness, and, in severe cases, neonatal mortality (*57*–*60*). In model organisms ranging from yeast to mice, complete loss of *Slc25a26* is incompatible with life and causes profound metabolic defects, highlighting a conserved requirement for mitoSAM (*38*, *60*–*62*).

Our data reveal a layer of mitochondrial metabolic regulation that operates upstream of transcriptional changes and respiratory capacity. Although OXPHOS and TCA cycle activity are usually tightly coupled, a selective restriction of a shared mitochondrial cofactor can uncouple these processes in vivo. In this scenario, mitoSAM availability acts as a permissive factor, enabling mitochondria to support both oxidative ATP synthesis and TCA-linked anabolic flux under increased metabolic demand. Cardiomyocytes are known to contain distinct mitochondrial subpopulations, including subsarcolemmal, interfibrillar, and perinuclear mitochondria, which may differ in metabolic traits (*63*, *64*). Our measurements reflect mitoSAM levels in bulk mitochondrial fractions, so we cannot rule out the possibility that individual cells or mitochondrial subpopulations maintain separate mitoSAM pools.

Loss of lipoylation impairs OGDHc activity, leading to αKG accumulation and depletion of aspartate and nucleotide pools. This metabolic profile contrasts with previous studies showing that elevated αKG and aspartate availability promote cardiomyocyte proliferation and hypertrophic growth (*19*, *23*, *65*). Instead, αKG accumulation in *Slc25a26*-deficient hearts indicates restricted TCA cycle flux rather than increased anabolic capacity. Although αKG can influence chromatin via αKG-dependent dioxygenases (*66*–*68*), we did not observe changes in global 5hmC levels, implying that the increase in cardiomyocyte cell–cycle activity mainly results from metabolic constraints rather than widespread epigenetic remodeling. While local epigenetic effects might occur, our data support a model in which impaired carbon flux and nucleotide depletion create a dual bioenergetic and biosynthetic burden during a period when cardiomyocytes normally exit the cell cycle and undergo terminal differentiation (*12*).

Notably, the onset of metabolic impairment in *Slc25a26*-deficient hearts coincided with the suckling-to-weaning transition, a period characterized by declining availability of medium-chain fatty acids and increased reliance on carbohydrate-derived substrates. This physiological shift likely exacerbates the intrinsic lipoylation defect by reducing octanoate availability for LA synthesis and increasing demand for pyruvate- and glutamate-derived carbon to enter the TCA cycle. Dietary supplementation with MCTs partially mitigated this transition, stabilizing metabolic homeostasis, reducing aberrant cardiomyocyte cell–cycle activity, and extending survival. Although the timing and nutritional composition of postnatal adaptation differ substantially between mice and humans, both species undergo marked metabolic remodeling after birth to meet rising cardiac energy demands. We therefore propose that the specific timing may differ, but the requirement to coordinate mitochondrial cofactor availability with metabolic demand is likely a conserved feature of cardiac adaptation.

Together, our data identify mitoSAM-dependent lipoylation as a stage-specific metabolic vulnerability that limits cardiac adaptation during postnatal maturation. This interpretation is supported by recent work showing that *Slc25a26* deletion in the adult heart is tolerated under resting conditions but reveals susceptibility under hemodynamic stress (*69*), highlighting the context-dependent requirement for mitoSAM-dependent metabolism. Similar periods of increased metabolic sensitivity are observed during the postnatal maturation of other organs, including the liver, brain, and kidney (*70*–*74*). Collectively, our findings suggest that mitochondrial cofactor availability represents a broader mechanism by which metabolic states gate developmental progression and tissue-specific vulnerability during periods of rapid physiological transition.

## MATERIALS AND METHODS

### Mouse breeding

*Slc25a26^LoxP/LoxP^* mice described earlier (*38*) were crossed with *Ckmm*-*NLS*-*Cre* mice (*40*). All mice used in these experiments were of C57BL/6N genetic background. Mice were housed in individually ventilated cages at a constant room temperature of 22° to 24°C, with free access to food and water, and were maintained on a 12-hour:12-hour light:dark cycle. Groups included both male and female animals.

A diet enriched with MCT (E15116-347, ssniff Spezialdiäten GmbH), supplying 56 kJ% fat, 27 kJ% carbohydrates, and 17 kJ% protein, was introduced on P11. The fat component comprised 30% hydrogenated coconut oil (C8 content 1.77%). MCT pellets were placed in a petri dish on the cage floor and replaced every 2 days. Breeding pairs had unlimited access to standard chow, which was provided on a wire rack above the cage bedding. Animal studies were approved by the local animal welfare ethics committee (Stockholm Ethical Committee) and conducted in accordance with national and European law.

### Genotyping primers for *Slc25a26*^*LoxP*^ alleles

*Slc25a26^LoxP^* primers are as follows: *SamcLox*_72_Fw 5′-ACA GCT GAG TGC AGT AGA GAC C-3′; *SamcLox*_72_Rv 5′-CCA TAA GAG ATA AGG CAC AGA GG-3′. *SamcLox*_73alt_Rv 5′-CTG GTG TGG CTT TAA CAT ACC T-3′. *Cre* primers are the following: Fw 5′-CAC GAC CAA GTG ACA GCA AT-3′; Rv 5′-AGA GAC GGA AAT CCA TCG CT-3′.

### Tissue collection, sample preparation, and animal procedures

Blood triglyceride levels were measured using an Accutrend Plus device (Roche Diagnostics) with Accutrend triglycerides test strips (Roche Diagnostics). After euthanasia by cervical dislocation or decapitation for animals younger than P10, dissected organs were immediately snap-frozen in liquid nitrogen and then used for proteome and metabolome analyses, and for genomic DNA (gDNA), mRNA, and protein extraction. Crude mitochondrial isolation for respiratory chain enzyme activity assays and ex vivo ATP production rates was performed on freshly dissected organs. Tissues for histological analyses were fixed in 10% (v/v) neutral-buffered formalin overnight at 4°C; the following day, tissues were transferred to 70% (v/v) ethanol and stored at 4°C until further use. All animal experiments received approval from the local animal welfare committee. All procedures adhered to institutional, national, and European guidelines and to the standards of the Federation of European Laboratory Animal Science Associations.

### Transthoracic echocardiography

Cardiac function was noninvasively monitored by transthoracic echocardiography using a high-resolution imaging system (Philips HDI 5000) with a 7- to 15-MHz CL 15–7 scanning head, as previously described (*75*, *76*). Briefly, 12-day-old mice were anesthetized with isoflurane (2%) and placed on a heating pad (37°C). Chest hair was removed with depilatory cream. After acquiring echocardiographic images, the mice were kept on a heating pad (37°C) without anesthesia until they recovered. M-mode imaging of the left ventricular short axis was analyzed, and cardiac contractility was expressed as a percentage of left ventricular fractional shortening using the formula: (*LVd* − *LVs*) / *LVd* * 100, where *LVd* and *LVs* represent diastolic and systolic left ventricular dimensions, respectively. Heart rate, posterior wall, and interventricular septum thickness were also measured.

### EdU administration

The Click-iT EdU Cell Proliferation Kit (Thermo Fisher Scientific) was used according to the manufacturer’s instructions to identify proliferating cells in vivo. Briefly, mice were injected intraperitoneally with EdU in saline (20 mg per kg of body weight) and kept alive for 4 hours to allow in vivo labeling. Afterward, mice were euthanized as usual; the heart and duodenum (positive control for EdU incorporation) were dissected and fixed overnight at 4°C in 10% normal-buffered formalin. Paraffin embedding and sectioning (10 μm) were performed according to standard procedures. Each slide included sections of the heart and duodenum. Sections were deparaffinized in xylene and rehydrated through a series of alcohol concentrations and water. The Click-iT chemistry reaction was performed according to the manufacturer’s instructions. After the Click-iT chemistry reaction, the sections were further processed for immunofluorescent staining.

### Immunofluorescent staining of heart sections

Formalin-fixed hearts were paraffin-embedded and sectioned at 10 μm. Deparaffinization was performed in xylene, followed by rehydration through graded alcohol concentrations and water. After the EdU click-iT chemistry reaction, sections were blocked in 10% normal goat serum in phosphate-buffered saline (PBS) for 30 min and then incubated overnight at 4°C with cardiac troponin T antibody (Invitrogen #MA5-12960) or pH3 (Ser^10^) antibody (Sigma-Aldrich #06-570). The following day, sections were washed and incubated with goat anti-mouse immunoglobulin G (IgG) secondary antibody (Invitrogen #A11017) or goat anti-rabbit IgG secondary antibody (Invitrogen #A-21072) for 2 hours at room temperature. Sections were then washed, and nuclei were stained with 4′,6-diamidino-2-phenylindole for 5 min at room temperature. WGA conjugated to a fluorophore (Invitrogen) was applied for 2 hours at room temperature.

### Transmission electron microscopy and histology

Formalin-fixed hearts were paraffin-embedded, sectioned at 10 μm, deparaffinized, and rehydrated according to standard protocols. Sections were stained with Mayer’s hematoxylin and eosin and picrosirius red, following standard protocols. Embedding, sectioning, staining, and image acquisition were performed by the Karolinska Institutet Unit for Morphological Phenotype Analysis. For transmission electron microscopy, freshly isolated hearts were cut into 1- to 2-mm^2^ pieces and fixed in 2% (v/v) formaldehyde/2% (v/v) glutaraldehyde in 0.1 M cacodylic acid for at least 48 hours at 4°C. Afterward, samples were rinsed in 0.1 M cacodylate buffer and postfixed with 2% osmium tetroxide in 0.1 M cacodylate buffer for 2 hours at 4°C. Samples were dehydrated through an ascending ethanol series, followed by stepwise acetone/LX-112 infiltration, and embedded in LX-112. Samples were postfixed, embedded, sectioned, and imaged by Karolinska Institutet Electron Microscopy Unit (EMil).

### Crude mitochondria isolation

Dissected hearts or quadriceps were washed in ice-cold 0.01 M PBS and cut into small pieces. The pieces were transferred to an isolation buffer [100 mM sucrose, 50 mM KCl, 1 mM EDTA, 20 mM *N*-tris(hydroxymethyl)methyl-2-aminoethanesulfonic acid, and 0.2% (w/v) bovine serum albumin (BSA, pH 7.2), supplemented with subtilisin A from *Bacillus licheniformis*] and homogenized with 20 strokes at 1000 rpm (Schuett homogen^plus^). Homogenates were differentially centrifuged at 800 and 8500 relative centrifugal force (rcf). Mitochondrial fractions were resuspended in the isolation buffer without BSA. Protein concentration was determined using the Qubit Protein Assay (Thermo Fisher Scientific).

### Blue native electrophoresis and in-gel activity assay

Ten micrograms of mitochondria was lysed with 4% digitonin to resolve supercomplexes. BN-PAGE was performed using the NativePAGE Novex bis-tris Gel System (Life Technologies) according to the manufacturer’s instructions. In-gel activity assays for complexes I and IV were performed as previously described (*77*).

### Isolated respiratory chain enzyme activities

ETC enzyme complex and citrate synthase activities in isolated mouse heart mitochondria were measured as previously described (*78*). All ETC activities were normalized to the citrate synthase activity in the mitochondrial suspension.

### Ex vivo ATP production rates

Ex vivo ATP production rates were measured as described previously (*78*). Freshly isolated mitochondria from mouse hearts were immediately resuspended in buffer [250 mM sucrose, 15 mM KH_2_PO_4_, 2 mM magnesium acetate, 0.5 mM EDTA, and BSA (0.5 g/liter, pH 7.2)]. Ten microliters of resuspended mitochondria was added to each well of a p96 plate containing a luciferase/luciferin-based ATP monitoring reagent (BioThema SL) and various substrate combinations: 15 mM glutamate + 15 mM succinate; 30 mM glutamate + 20 mM malate; 30 mM pyruvate + 10 mM malate; 20 mM αKG; 20 mM succinate + rotenone (1 mg/liter); 20 μM decanoyl-l-carnitine + 1 mM malate; 10 μM lauryl-l-carnitine + 1 mM malate; 5 μM myristoyl-l-carnitine + 1 mM malate; and 5 μM palmitoyl-l-carnitine + 1 mM malate. The reaction was initiated by injecting 15 μl of ADP to a final concentration of 0.6 mM. Luminescence was monitored over 7 min using a Tecan SPARK microplate reader at a constant temperature of 25°C. Subsequently, an ATP internal standard (final concentration of 0.5 μM) was added to each well, and the increase in luminescence was used to convert counts into ATP μmol. Each substrate was measured in technical triplicates. Results were expressed in units (micromole ATP per minute), normalized to citrate synthase units. The content of the microplate wells, with a final volume of 250 μl, included the following: 150 mM sucrose, 15 mM K_2_HPO_4_, 2 mM MgAc_2_, 0.5 mM EDTA, firefly luciferase, d-luciferin (0.1 g/liter), l-luciferin (0.004 g/liter), BSA (1 g/liter), 1 μM Na_2_P_2_O_7_, 0.6 mM ADP, 1 mM AMP, 0.2 μM DAPP, and 0.5 μM ATP (added as an internal standard at the end of the measurements).

### CoQ_9_ and CoQ_10_ levels measurement

CoQ_9_ and CoQ_10_ levels were measured as previously described (*79*). Briefly, isolated mitochondria were resuspended in 200 μl of 1 mM CuSO4, and then 200 μl of ethanol was added. Samples were ultrasonicated at high power for 5 min in an ice-cold water bath (Bioruptor, Diagenode). After adding 400 μl of hexane, the samples were vortexed and centrifuged at 2500 rcf for 5 min at 4°C. The hexane-soluble upper fraction was collected and dried using a vacuum concentrator (Eppendorf). CoQ9 and CoQ10 levels were normalized to protein content.

### SAM and SAH levels

SAM and SAH levels were measured as previously described (*38*). In brief, metabolites were extracted from mitochondrial pellets or total homogenates using ice-cold 80% (v/v) methanol spiked with the labeled internal standards SAM[^2^H_3_] and SAH[^2^H_4_], followed by incubation on ice. The extracts were analyzed using a Waters XEVO TQ-XS (Milford, USA). Data acquisition and peak integration were performed using MassLynx 4.1 software (Waters). SAM and SAH levels were normalized to protein content.

### Untargeted metabolomics of mouse hearts

Samples were stored at −80°C until processing. Metabolites were homogenized in precooled (4°C) 80% acetonitrile (ACN) using a Qiagen TissueLyser III system. Homogenates were centrifuged at 18,000 rcf for 15 min at 4°C, and the supernatant was transferred to a fresh tube. Residual protein content was quantified using the Pierce BCA Protein Assay (Thermo Fisher Scientific), and all samples were normalized to 0.1 μg of protein/μl in 80% ACN.

Metabolites were analyzed by LC-high resolution MS/MS as previously described (*80*, *81*). Briefly, extracts were loaded onto a QExactive HF-X mass spectrometer coupled to a Vanquish Flex LC system (Thermo Fisher Scientific, Bremen, Germany). Metabolites were chromatographically resolved on a Millipore SeQuant ZIC-pHILIC column using a binary gradient of 10 mM ammonium acetate in water at pH 9.8 and ACN. Precursor and product ion spectra of metabolites were acquired in both positive and negative polarities. Metabolites were quantified in Compound Discoverer 3.3 by searching against an in-house spectral library of previously validated metabolites. Principal components analyses, pathway enrichment, and statistical analyses were performed using MetaboAnalyst 6.0 (*82*).

### RNA isolation and quantitative PCR with reverse transcription

Hearts were stored at −80°C until processing. Tissue was homogenized using a Fastprep-24 tissue homogenizer (MP Biomedicals) with 2 × 30 s at 6000 rpm in TRIzol (Thermo Fisher Scientific). mRNA was extracted using a standard TRIzol/chloroform extraction method. RNA concentrations were measured using a Nanodrop ND-1000 (Thermo Fisher Scientific). Isolated RNA was treated with deoxyribonuclease (DNA-free Kit, Ambion) and subsequently reverse transcribed using the High-Capacity cDNA Reverse Transcription Kit (Applied Biosystems). Quantitative reverse transcription polymerase chain reaction (PCR) was performed on a QuantStudio 6 system using TaqMan probes and TaqMan Universal Master Mix II with uracil-*N*-glycosylase (Thermo Fisher Scientific). Normalization was carried out to Hprt, Actb, or 18*S*. The relative expression of mRNAs was determined using the comparative method (2-∆∆Ct). TaqMan probes are listed in table S1.

### SDS-PAGE and immunoblot analysis

Samples were stored at −80°C until processing. Heart and quadriceps tissues were homogenized using the Qiagen TissueLyser III system in T-PER Tissue Protein Extraction Reagent (Thermo Fisher Scientific), containing a protease inhibitor cocktail (Roche) and a phosphatase inhibitor cocktail (PhosSTOP, Roche). Protein concentration was measured using the Pierce BCA Protein Assay (Thermo Fisher Scientific). Protein lysates (20 to 30 μg) were resuspended in NuPAGE LDS Sample Buffer (Thermo Fisher Scientific), incubated at 70°C for 10 min, and loaded onto precast Bolt 4 to 12% or 12% Bolt bis-tris gels (Thermo Fisher Scientific) in an XCell SureLock electrophoresis system (Thermo Fisher Scientific). After electrophoresis, proteins were transferred to nitrocellulose or polyvinylidene difluoride membranes, blocked with EveryBlot Blocking Buffer (Bio-Rad) for 5 min at room temperature or with 5% fat-free milk in tris-buffered saline with Tween 20 (TBST) for 1 hour, and immunoblotted with primary antibodies. Primary antibodies were incubated overnight at 4°C, followed by secondary antibodies, anti-mouse IgG (Cytiva, #NA9310) or anti-rabbit IgG (Cytiva, #NA9340), conjugated to horseradish peroxidase (HRP) for 1 hour at room temperature. Clarity Western ECL Substrate (Bio-Rad) was used for detection. Immunoblot images were captured with ChemiDoc XRS+ (Bio-Rad). If necessary, nitrocellulose membranes were stripped with Restore Western blot Stripping Buffer (Thermo Fisher Scientific). The primary antibodies used included as follows: rodent OXPHOS cocktail (Abcam, #ab110413), vinculin (CST, #4650), HSC70 (Santa Cruz Biotechnology, #sc-729), α-tubulin (Millipore, #CP06), glyceraldehyde-3-phosphate dehydrogenase (Abcam, #ab8245), TOMM20 (Sigma-Aldrich, #HPA011562), DLAT (Proteintech, #13426-1-AP), DLST (Sigma-Aldrich, #HPA003010), OGDHE1 (Sigma-Aldrich, #HPA020347), citrate synthase (Santa Cruz Biotechnology, #sc-390693), aconitase-2 (Abcam, #ab110321), LA (Millipore, #437695), ACC2 (CST, #3662), phospho-ACC2 (Ser^79^) (CST, #3661), NDUFA9 (Molecular Probes, #A21362), SDHA (Abcam, #ab14715), and ATPA1 (Abcam, #ab14748). Uncropped images are available in the extended file data S4.

### Detection of 5hmC

Hearts were stored at −80°C until processing. gDNA was isolated using the high-salt chloroform method (*83*). The concentration was determined using a Nanodrop ND-1000 (Thermo Fisher Scientific), and all samples were diluted to 50 ng/μl. One hundred microliters of gDNA was mixed with an equal volume of DNA denaturing buffer (200 mM NaOH and 20 mM EDTA) and denatured at 95°C for 10 min, followed by immediate cooling on ice for 5 min. Samples were mixed with 200 μl of 20× saline-sodium buffer [3 M NaCl and 0.3 M sodium citrate (pH 7.4)] and brought up to 500 μl with nuclease-free water. A twofold serial dilution was prepared. A positively charged Hybond-N+ nylon membrane (Cytiva) was prewetted in 2× saline-sodium buffer for 10 min and assembled in a dot blot apparatus (Carl Roth). One hundred microliters of each dilution was loaded into individual wells and allowed to filter through the membrane under a gentle vacuum. The dot blot apparatus was disassembled; the membrane was air-dried for 5 min and ultraviolet cross-linked for 30 s at 120,000 mJ/cm^2^. The membrane was blocked in EveryBlot Blocking Buffer (Bio-Rad) for 5 min at room temperature and incubated with anti-5hmC antibody (Active Motif, #39769-AF) for 1 hour at room temperature. Afterward, the membrane was washed three times with TBST buffer, followed by incubation with the secondary anti-rabbit antibody (Cytiva, #NA9340) conjugated to HRP for 1 hour at room temperature. Clarity Western ECL Substrate (Bio-Rad) was applied. Immunoblot images were acquired with ChemiDoc XRS+ (Bio-Rad).

### Sample preparation for proteomics

Hearts were stored at −80°C until processing. Samples were cryogenically pulverized using the Covaris CryoPREP system (Covaris, Woburn, MA, USA). Two hundred microliters of lysis buffer [4% SDS, 50 mM Hepes (pH 7.6), and 1 mM dithiothreitol (DTT)] was added, and samples were heated to 95°C and sonicated with an Ultrasonic Processor UP200St (Hielscher Ultrasonics, Teltow, Germany). Lysates were centrifuged, and supernatants were collected for digestion. For each sample, 200 μg of protein was reduced for 30 min in lysis buffer at 37°C and then alkylated with 10 mM chloroacetamide for 10 min at room temperature in the dark. Protein cleanup and digestion were performed using the SP3 method (*84*, *85*). Briefly, proteins were bound to SeraMag SP3 bead mix (40 μl) in 70% ACN and then washed twice with 70% ethanol and once with 100% ACN. The beads-protein mixture was reconstituted in 100 μl of digestion buffer containing Rapizyme Trypsin (MS grade, Waters) [50 mM Hepes (pH 7.6), 10% ACN, 10 mM CaCl_2_, and 0.4 μg Rapizyme] and incubated overnight at 37°C. The following day, peptides were recovered by >95% ACN precipitation, washed, and resuspended in 0.1% formic acid for LC-MS injection.

### LC–electrospray ionization–MS/MS (DIA, timsTOF HT)

Five hundred milligrams of peptides was loaded onto Evotip PURE (EvoSep). Separation was performed on an Evosep One system using a 30–samples/day gradient on a PepSep FIFTEEN column (15 cm by 150 μm, 1.5 μm; Bruker) at 40°C. Mobile phases were: A = 0.1% Formic acid (FA) in water, B = 0.1% FA in ACN. Eluted peptides were analyzed on a timsTOF HT mass spectrometer (Bruker) equipped with a CaptiveSpray source. Data were acquired in dia-PASEF mode with a mass/charge ratio (*m*/*z*) range of 300 to 1300 and ion mobility 0.6 to 1.6 1/*K_0_*. Isolation windows were optimized with Py-DiaID (*86*). Collision energies were ramped from 15 to 70 eV, with a total cycle time of 1.8 s.

The separation column was a PepSep FIFTEEN 15 cm by 150 μm by 1.5 μm (Bruker 1893474) connected to a 20 μM ZDV Sprayer (Bruker 1865710) at 40°C. Mobile phase A was 0.1% FA in MQ, and B was 0.1% FA in ACN. Online LC-MS was performed on a timsTOF HT mass spectrometer (Bruker) with the CaptiveSpray source, capillary voltage 1500 V, dry gas flow of 3 liters/min, and dry gas temperature at 180°C. Collision energy was set to 20 eV for 1/*K_0_* 0.60 Vs/cm and 59 eV for 1/*K_0_* 1.60 Vs/cm^2^. Data were acquired using Timscontrol v 6.0.6 and Compass HyStar 6.3.1.8. The above conditions were used for data-independent acquisition (DIA) diaPASEF (parallel accumulation-serial fragmentation). For diaPASEF analysis, precursor ions were selected in the range of 300 to 1300 *m*/*z* and 0.6 to 1.6 1/*K_0_* IM. Optimized isolation windows were based on the Bruker human library, with assistance from the Py-DiaId Python package (*86*). Collision energies for fragmentation were ramped from 15 to 70 eV. Parallel accumulation and serial fragmentation were performed with a cycle time of 1.8 s.

### Proteomic data analysis (DIA)

Raw DIA files were processed in Spectronaut (Biognosys) using the DirectDIA workflow for label-free quantification. A project-specific spectral library was generated directly from the DIA data. Search parameters: 1% false discovery rate (FDR) at the peptide and protein level, up to two missed cleavages, enzyme specificity = trypsin (*87*). Searches were performed against the UniProt mouse proteome (ID: 10088). Protein quantification was based on the top three peptides per major group and the top six per minor group, using only unique peptides mapped to a single gene ID.

### Downstream statistical analysis of proteomics data

MS2-based protein intensity values exported from Spectronaut (DIA datasets), Proteome Discoverer (TMT datasets), or MaxQuant (LFQ datasets) were processed in R (v4.5.1). Protein-level expression analysis followed the standard DEP2 workflow (*88*). Briefly, data were normalized using normalize_vsn() (variance-stabilizing transformation), and differential abundance was tested using test_diff(). Proteins were considered significantly altered at adjusted *P* < 0.05 and |log_2_FC| > 0.58, unless otherwise indicated. Functional enrichment analysis was performed with clusterProfiler (*89*), applying Gene Ontology, KEGG pathway, and Reactome terms. Enrichment results were visualized using clusterProfiler plotting functions and ggplot2.

### Sample preparation for redox proteomics

Hearts were stored at −80°C until processing. Samples were thawed on ice, minced, and transferred to tubes containing 400 μm of LoBind silica beads and 100 μl of lysis buffer [4 M urea, 0.1% ProteaseMAX (Promega), and 50 mM NaCl in 100 mM tris-HCl (pH 8.5)]. Samples were homogenized with a Disruptor Genie (2800 rpm, 2 min cycles, repeated ×5 with cooling) and centrifuged (14,000 rcf, 10 min, 4°C). Supernatants were collected, sonicated (40 s, 20% amplitude, 2 s on/2 s off), and protein concentrations were measured with Pierce BCA Protein Assay (Thermo Fisher Scientific).

For redox labeling, 50 μg aliquots were adjusted to 20 mM 4-(2-hydroxyethyl)piperazine-1-propanesulfonic acid (EPPS) buffer at pH 8.5 and alkylated with 50 mM iodoacetamide for 1 hour at 25°C in the dark. Proteins were precipitated (chloroform/methanol), resuspended in 8 M urea, sonicated, and diluted in EPPS buffer at pH 7.2. Oxidized cysteines were reduced with 0.5 mM DTT (45 min, 25°C) and realkylated with 100 mM *N*-ethylmaleimide (2 hours, 25°C, dark). Proteins were reprecipitated, resuspended, and digested with trypsin (1 μg, 6 hours, 25°C). Peptides were cleaned on a HyperSep C18 plate (Thermo Fisher Scientific) and dried by vacuum centrifugation.

### LC-MS/MS (Data-Dependent Acquisition, Orbitrap Q Exactive HF)

Peptides (∼2 μg) were injected onto an EASY-Spray C18 column (50 cm, Thermo Fisher Scientific) coupled to an Ultimate 3000 nanoUPLC (Thermo Fisher Scientific). Separation was achieved with a 90-min gradient (4 to 26% B in 90 min, 26 to 95% B in 5 min; flow 300 nl/min). Mobile phase A: 0.1% FA in water; B: 0.1% FA in 98% ACN.

MS acquisition was performed on a Q Exactive HF (Thermo Fisher Scientific) with full MS scans from *m*/*z* 375 to 1500 at *R* = 120,000, automatic gain control (AGC) target 5 × 10^6^, max injection time 80 ms. Data-dependent MS/MS of the top 18 precursors (charge 2 + −7+) was performed with higher-energy collisional dissociation (33% normalized collision energy), *R* = 60,000, AGC target 2 × 10^5^, max IT 54 ms, isolation width 1.4 Th, and 45 s dynamic exclusion.

### Redox proteomics downstream data analysis

Raw data files are processed in Proteome Discoverer v3.0 (Thermo Fisher Scientific) using the Mascot v2.5.1 search engine. The database used was SwissProt mouse proteome. The search parameters were set to two missed cleavages, precursor mass tolerance 10 parts per million (ppm), and fragment tolerance 0.02 Da. Fixed modifications are as follows: carbamidomethylation and *N*-ethylmaleimide (Cys), and TMTpro (Lys, N terminus). Variable modifications are the following: Met oxidation, Asn/Gln deamidation. FDR ≤5% at the peptide level using Percolator. Downstream statistical analyses were performed in R using limma. (*90*).

### Single-cell RNA-seq data analysis

Single-cell RNA-seq data from murine heart were obtained from the Tabula Muris dataset (*34*) in Seurat object format. Data processing and visualization were carried out in R (version 4.5.1) using the Seurat package (*91*). Standard preprocessing steps included normalization with SCTransform(), dimensionality reduction with RunPCA(), RunUMAP(), and RunTSNE(), followed by neighborhood graph construction with FindNeighbors() and clustering with FindClusters(). Cell-type annotation of the resulting clusters was performed with the clustermole package (https://igordot.github.io/clustermole/). Visualization and plotting were carried out with Seurat functions and ggplot2.

### Immunofluorescent image capture and quantification

Immunofluorescence-stained sections were imaged on a confocal laser-scanning microscope (Leica Stellaris 5 X, Leica Microsystems). Z-stack images were acquired in Nyquist-sampled sequential mode. Leica Application Suite X (Leica Microsystems) and Fiji (ImageJ) were used to uniformly adjust contrast and brightness. For cardiomyocyte cross-sectional area, 900 cardiomyocytes per heart section were manually traced, and their areas were measured in Fiji. The number of EdU^+^ and pH3^+^ cardiomyocytes per square millimeter of heart was assessed by counting EdU^+^ and pH3^+^ cardiomyocytes defined by cardiac troponin T and/or WGA staining within a region of interest in Fiji (ImageJ) and dividing by the area of the region of interest in square millimeters.

### High-resolution respirometry on permeabilized left ventricle myocardium

Left ventricular dissection, preparation, fiber-bundle permeabilization, and high-resolution respirometry were performed according to (*92*). The permeabilization step lasted 25 min at 4°C. O_2_ consumption was measured at 37°C in permeabilized fiber bundles (1 to 3 mg wet tissue weight) using the Oroboros O_2_K Oxygraph with MiR05 respiration buffer. After sample addition, chambers were hyperoxygenated to ∼450 nmol/liter. Subsequently, fatty acid oxidation was assessed using the substrate-uncoupler-inhibitor-titration protocol. Briefly, basal oxygen consumption was supported by palmitoyl-carnitine + malate (20 and 0.1 mM, respectively). Following stabilization, state 3 respiration was determined by adding ADP (2.5 mM). Glutamate and malate (10 and 2 mM, respectively) were added to determine fatty acid oxidation linked to complex I. Succinate (10 mM) was added to support electron flow through complexes I and II. Residual oxygen consumption was measured after adding antimycin A (2.5 mM) and subtracted from oxygen flux as a baseline correction. Mitochondrial membrane integrity was assessed in all experiments with cytochrome *c* (10 mM). O_2_ flux in chambers was normalized to wet tissue weight.

### Quantification, statistical analysis, and visualization

The number of mice analyzed per experiment is indicated in the corresponding figure legends. Statistical significance between two groups was assessed using Student’s *t* test and the Mantel-Cox log-rank test; one-way and two-way analyses of variance (ANOVAs) were used for multiple comparisons. Analyses were carried out in Prism 10 (GraphPad Software). The number of replicates and the specific statistical test are detailed in each figure legend and in Materials and Methods. Unless stated otherwise, values represent means ± SD. No statistical method was used to predetermine sample size. Figure preparation was carried out using Prism 10 (GraphPad Software) and Adobe Illustrator 2026 (Adobe).

### Data availability

The proteomics data generated in this study have been deposited in the ProteomeXchange via the jPOST repository (*93*) under the dataset identifiers JPST004144 and PXD070035 (https://proteomecentral.proteomexchange.org/ui?pxid=PXD070035). Raw redox proteomics data are deposited at ProteomeXchange via PRIDE (*94*) under the dataset identifier PXD050486 (https://proteomecentral.proteomexchange.org/ui?pxid=PXD050486).

Datasets used in this study include MitoXplorer v2 and FASTA files from UniProt (*Mus musculus*, September 2018). Source data are provided with this paper.
