# Supplementary material for: Loss of the mitochondrial SAM transporter reveals a lipoylation-dependent metabolic vulnerability in the postnatal heart
Source: Sci Adv. 2026 Aug 26;12(35):eaeg8792. doi: 10.1126/sciadv.aeg8792 (PMC13510680; doi:10.1126/sciadv.aeg8792)

Science Advances
AAAS

Supplementary Materials for

Loss of the mitochondrial SAM transporter reveals a lipoylation-dependent metabolic vulnerability in the postnatal heart

Anastasia Rumyantseva *et al.*

Corresponding author: Christoph Freyer, christoph.freyer@ki.se; Anna Wredenberg, anna.wredenberg@ki.se

Sci. Adv. **12**, eaeg8792 (2026)
DOI: 10.1126/sciadv.aeg8792

The PDF file includes:

Figs. S1 to S9
Table S1
Legends for data S1 to S3
Data S4

Other Supplementary Material for this manuscript includes the following:

Data S1 to S3

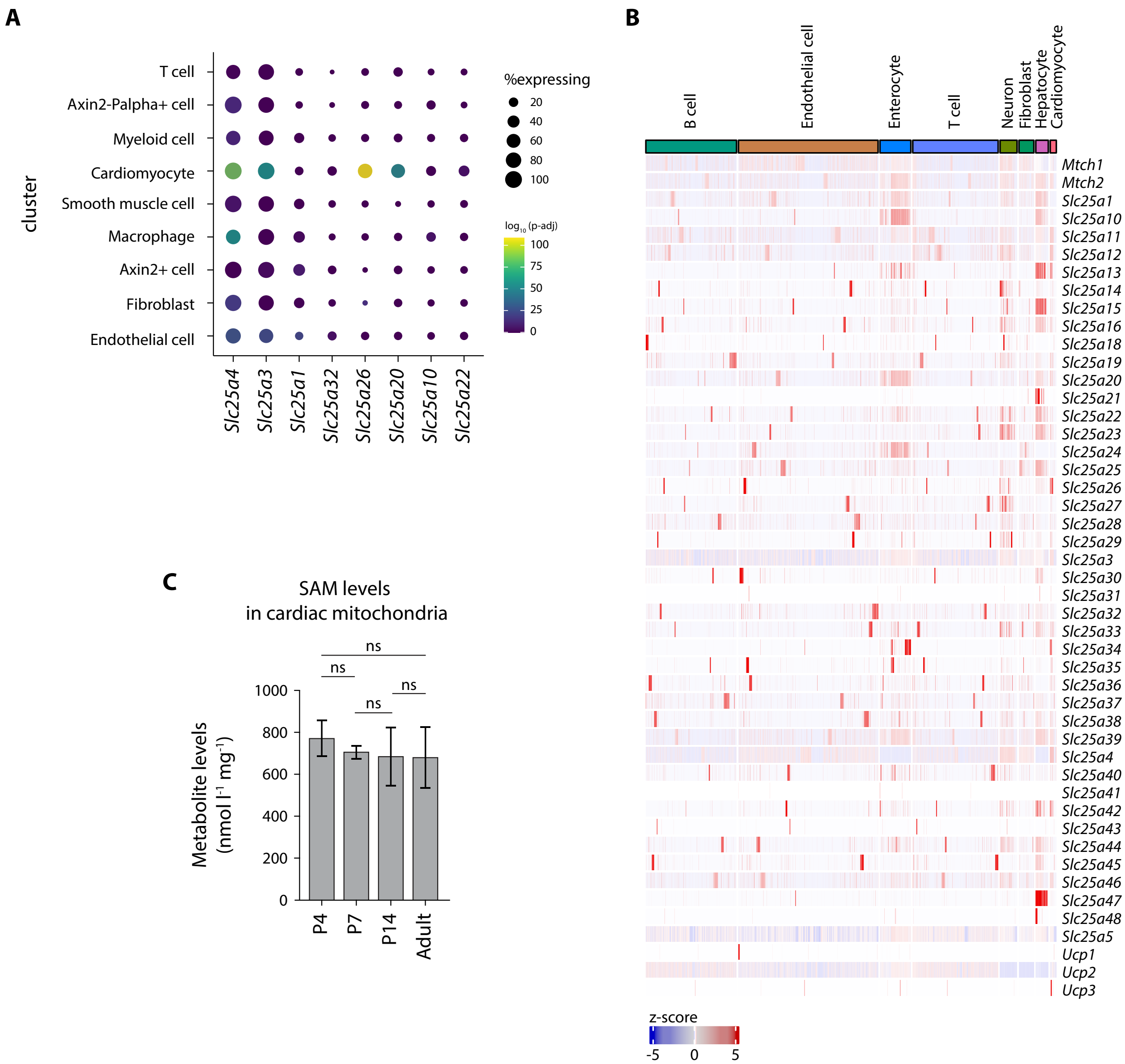

Fig. S1. *Slc25a26* expression is high in cardiomyocytes and mitoSAM levels are maintained during postnatal cardiomyocyte maturation.

(**A**) Dot plot of selected SLC25 family members in the indicated clusters. Data were obtained from a publicly available single-cell dataset (28). The size of the dots indicates the proportion of cells in the cluster expressing the indicated gene. Color indicates the significance of differential expression of the indicated gene. (**B**) Heat map showing expression of all SLC25A family members across the indicated lineages. (**C**) Targeted metabolic analysis of mitoSAM levels at the indicated time points in Ctrl cardiac mitochondria (n=5-8 per time point). Data are presented as mean ± SD; one-way ANOVA with Tukey's multiple-comparisons test; ns = not significant.

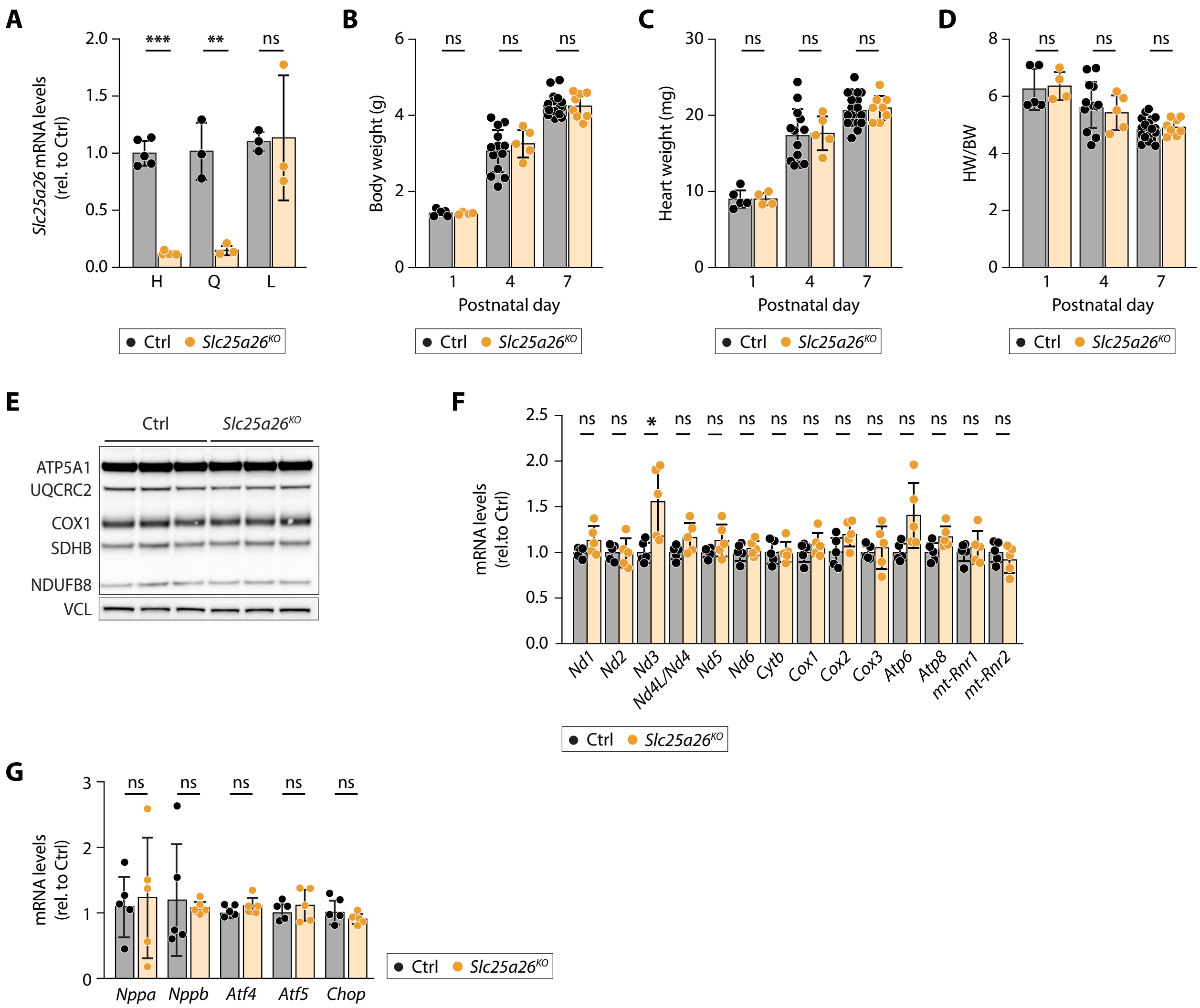

Fig. S2. *Slc25a26^{KO}* mice do not show growth retardation and mitochondrial gene expression defect at P7.

(**A**) Transcript levels of *Slc25a26* in heart (H), quadriceps (Q) and liver (L) in Ctrl and *Slc25a26^{KO}* mice, qRT-PCR (n=5 for heart, n=3 for quadriceps and liver per genotype). (**B**) Body weight of Ctrl and *Slc25a26^{KO}* mice at the indicated time points (n=4-19 per genotype and time point). (**C**) Heart weight of Ctrl and *Slc25a26^{KO}* mice at the indicated time points (n=4-19 per genotype and time point). (**D**) Heart weight (HW) normalized to body weight (BW) of Ctrl and *Slc25a26^{KO}* mice at the indicated time points (n=4-19 per genotype and time point). (**E**) Immunoblot of Ctrl and *Slc25a26^{KO}* heart lysates for the indicated ETC complex subunits at P7 vinculin (VCL) was used as a loading control (n=3 per genotype). (**F**) Transcript levels of the indicated mtDNA-encoded genes in Ctrl and *Slc25a26^{KO}* hearts at P7, qRT-PCR (n=5 per genotype). (**G**) Transcript levels of the indicated cardiomyopathy and stress-response markers in Ctrl and *Slc25a26^{KO}* hearts at P7,

qRT-PCR (n=5 per genotype). Data in (A-D, F and G) are presented as mean ± SD; each dot represents an individual mouse; two-tailed unpaired Student's t-test; ns = not significant, *$p < 0.05$, **$p < 0.01$, ***$p < 0.001$.

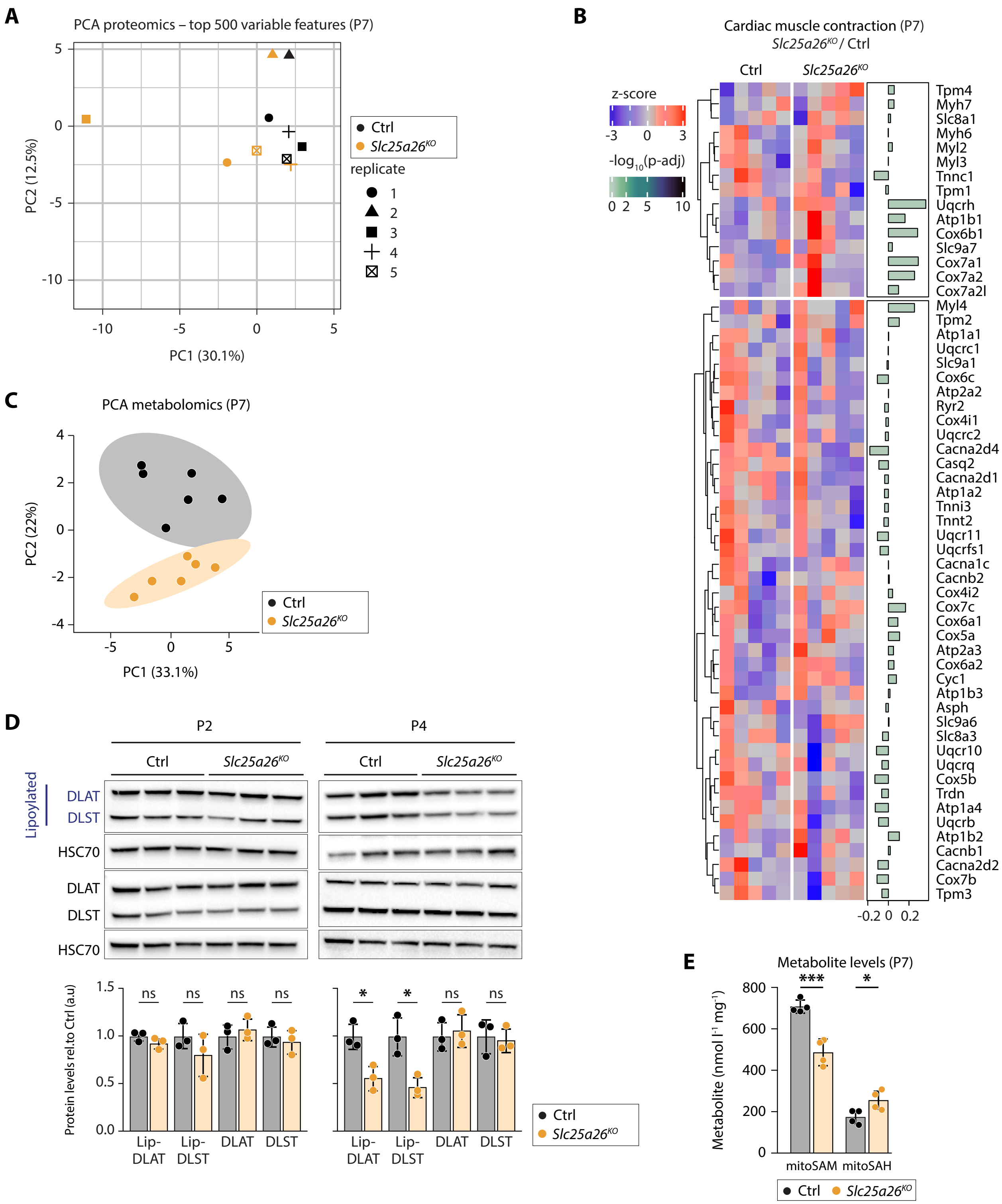

Fig. S3. Loss of *Slc25a26* selectively impairs lipoylation before global proteome changes. (**A**) Principal component analysis (PCA) of LC-MS/MS label-free whole-heart proteomics, Ctrl and *Slc25a26*^{KO} at P7 (n=5 per genotype). (**B**) Heat map of relative z-scores from heart proteomics data, showing proteins in the KEGG cardiac muscle contraction pathway in *Slc25a26*^{KO} hearts

compared with Ctrl at P7. (**C**) PCA of untargeted metabolomics data in Ctrl and *Slc25a26*^{KO} hearts at P7 (n=6 per genotype). (**D**) Immunoblot of Ctrl and *Slc25a26*^{KO} heart lysates, depicting lipoylated and apo-forms of DLAT and DLST at P2 and P4; HSC70 was used as the loading control (n=3 per genotype). Quantification is shown below. (**E**) Targeted metabolic analysis of mitoSAM and mitoSAH levels in Ctrl and *Slc25a26*^{KO} cardiac mitochondria at P7 (n=4 per genotype). Data in (E) are presented as mean ± SD; each dot represents an individual mouse. Two-tailed unpaired Student's t-test; ns = not significant, *p < 0.05. Statistical analysis for (B) was performed using a linear model with moderated t-statistics. P-values were adjusted for multiple testing (Benjamini-Hochberg).

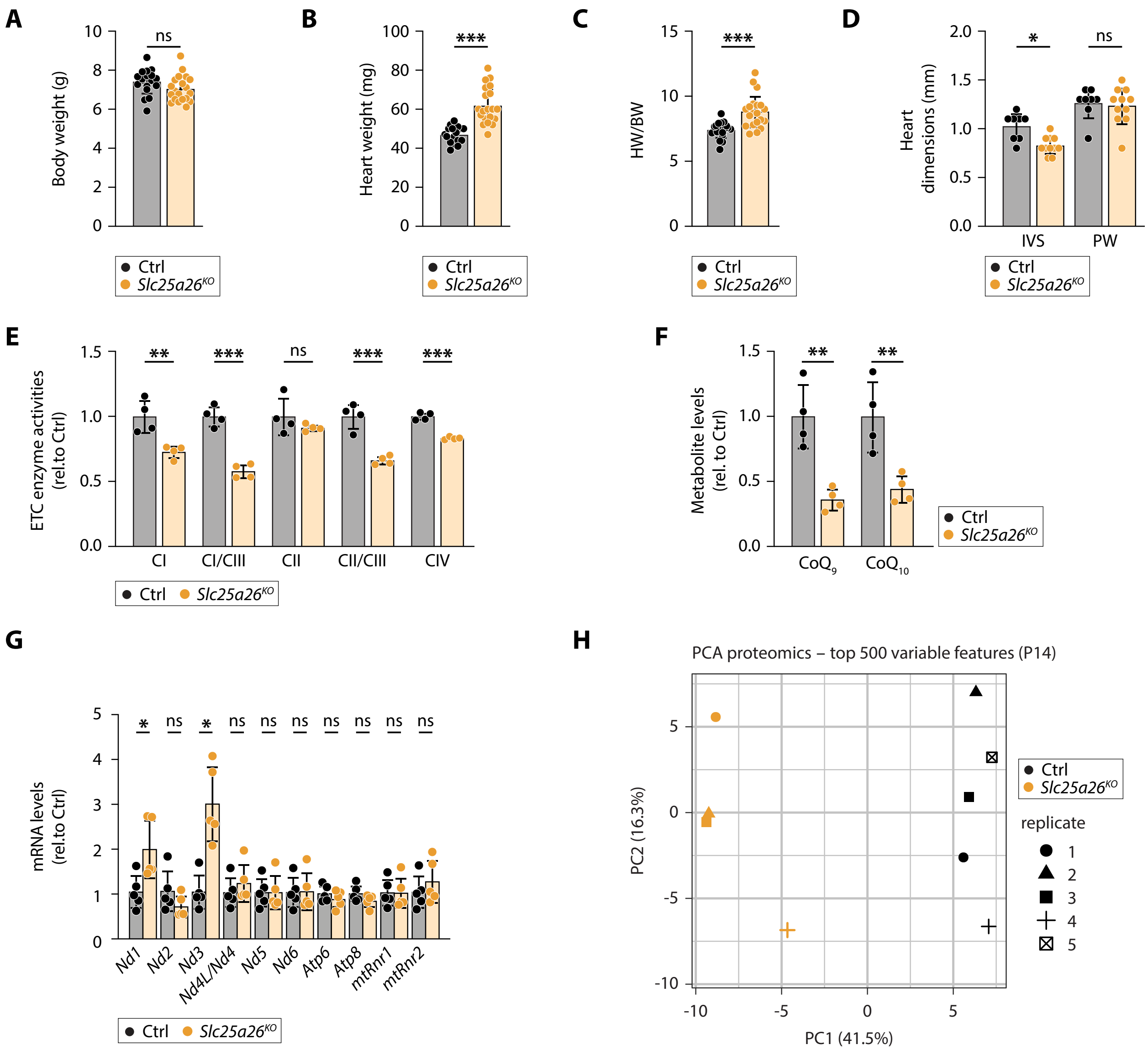

Fig. S4. *Slc25a26^{KO}* mice develop cardiomyopathy at P14, with moderate impairment of ETC function.

(**A**) Body weight of Ctrl and *Slc25a26^{KO}* mice at P14 (n=18 – 20 per genotype). (**B**) Heart weight of Ctrl and *Slc25a26^{KO}* mice at P14 (n=18 – 20 per genotype). (**C**) Heart weight (HW) normalized to body weight (BW) of Ctrl and *Slc25a26^{KO}* mice at P14 (n=18 – 20 per genotype). (**D**) Echocardiographic analysis of Ctrl (n=8 mice) and *Slc25a26^{KO}* mice (n=11 mice) at P12, showing dimensions of the intraventricular septum (IVS) and posterior wall (PW). (**E**) Relative isolated electron transport chain (ETC) enzyme activities in Ctrl and *Slc25a26^{KO}* cardiac mitochondria (n=4 per genotype) at P14. (**F**) Targeted metabolic analysis of CoQ9 and CoQ10 levels in Ctrl and *Slc25a26^{KO}* cardiac mitochondria at P14 (n=4 per genotype). (**G**) Transcript levels of the indicated

mtDNA-encoded genes in Ctrl and *Slc25a26*^{KO} hearts at P14, qRT-PCR (n=5 per genotype). (**H**) PCA of LC-MS/MS label-free whole-heart proteomics, Ctrl and *Slc25a26*^{KO} at P14 (n=5 for Ctrl, n=4 for *Slc25a26*^{KO}). Data in (A-G) are presented as mean ± SD; each dot represents an individual mouse; two-tailed unpaired Student's t-test; ns = not significant, *p<0.05, **p<0.01, ***p<0.001.

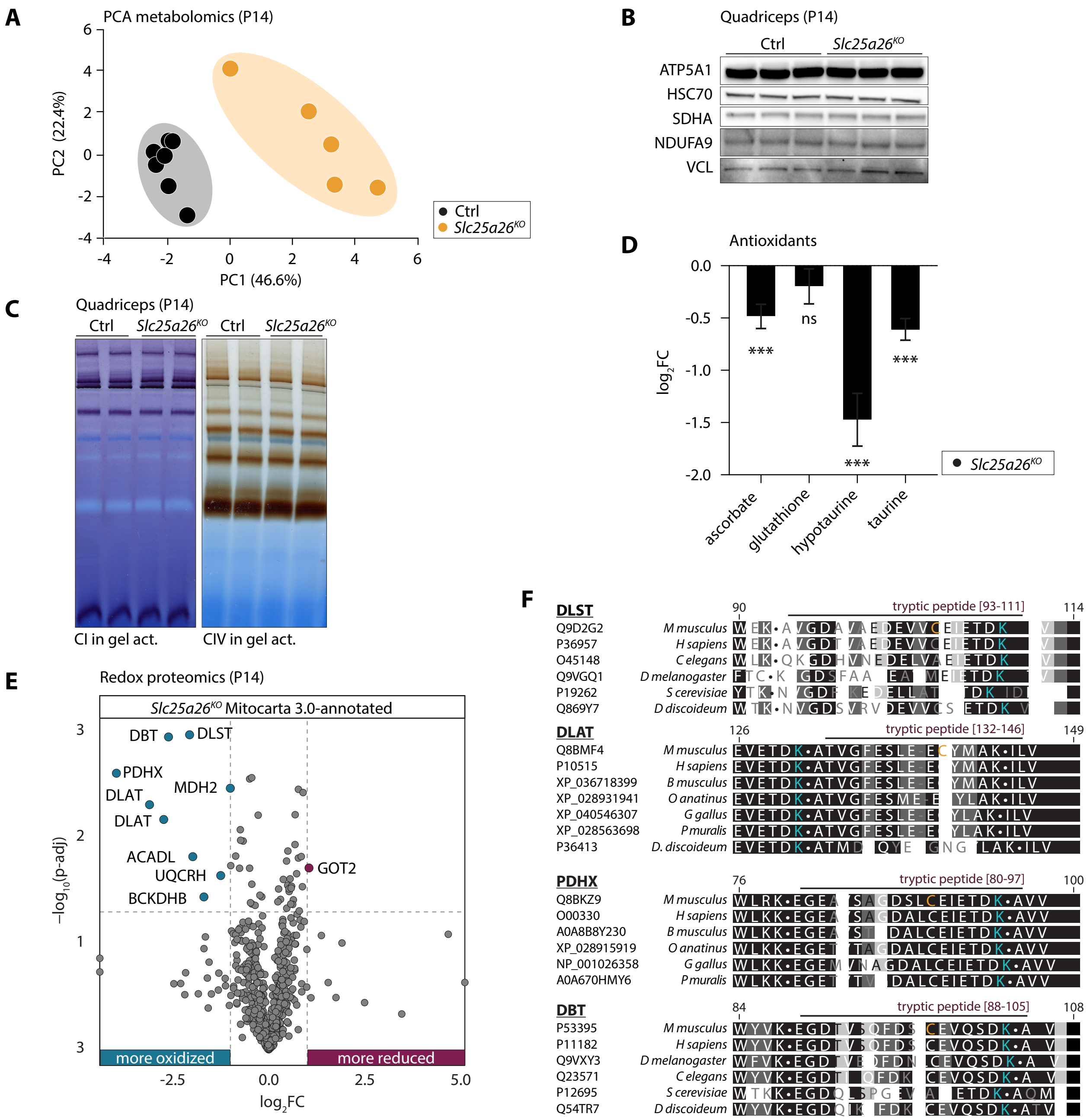

Fig. S5. Tissue-specific impact of Slc25a26 loss: ETC activity remains unperturbed in skeletal muscle, whereas the heart shows oxidized cysteines in the E2 subunits of 2-oxoacid dehydrogenase complexes.

(**A**) PCA of untargeted metabolomics data in Ctrl and *Slc25a26*^{KO} hearts at P14 (n=7 for Ctrl, n=5 for *Slc25a26*^{KO}). (**B**) Immunoblot of Ctrl and *Slc25a26*^{KO} quadriceps lysates, depicting the OXPHOS subunits ATP5A1 (complex V), SDHA (complex II), and NDUFA9 (complex I). HSC70 and vinculin (VIC) were used as loading controls (n=3 per genotype). (**C**) In-gel activity staining for CI and CIV in quadriceps digitonin-solubilized mitochondria at P14, separated by BN-

PAGE. (**D**) Log_2FC of antioxidants detected in untargeted metabolomics in *Slc25a26*^{KO} hearts compared with Ctrl at P14. (**E**) Volcano plot of the mitochondrial redox proteome from *Slc25a26*^{KO} (n=4) vs Ctrl (n=4) hearts at P14. Peptides containing differentially changed cysteines (FC, adjusted p-value < 0.05) are shown in purple (reduced) and blue (oxidized). (**F**) Protein sequence alignments of the 2-oxoacid dehydrogenase E2 subunits DLST, DLAT, pyruvate dehydrogenase protein X (PDHX), and dihydrolipoamide branched-chain transacylase (DBT). Mouse (*Mus musculus*) was used to align orthologs from humans (Homo sapiens), roundworm (*Caenorhabditis elegans*), fruit fly (*Drosophila melanogaster*), brewer's yeast (*Saccharomyces cerevisiae*), amoebae (*Dictyostelium discoideum*), blue whale (*Balaenoptera musculus*), platypus (*Ornithorhynchus anatinus*), chicken (*Gallus gallus*), and the common wall lizard (*Podarcis muralis*). Differentially oxidized cysteines in the mouse sequences are indicated in orange. Lipoylated lysines are shown in blue.

Data in (D) are presented as $log_2FC \pm$ s.e.m.; statistical significance was assessed using a two-tailed unpaired Student's t-test; ns = not significant, ***$p < 0.001$. Significance analysis for (E) was performed using a linear model with moderated t-statistics and a Benjamini-Hochberg multiple-testing correction.

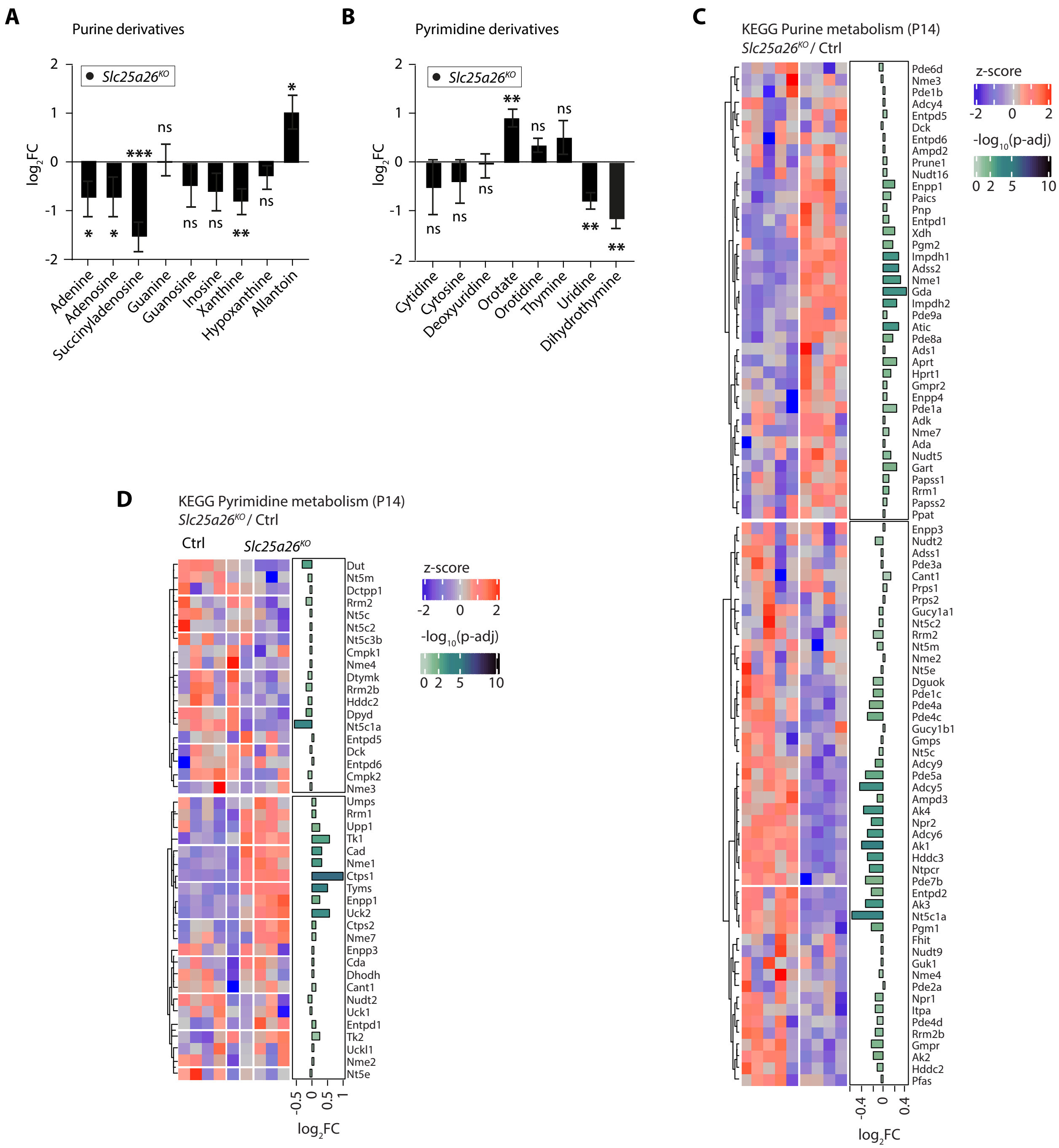

Fig. S6. *Slc25a26* deficiency leads to nucleotide depletion, despite upregulation of nucleotide biosynthetic enzymes.

(**A**) Log$_2$FC of purine derivatives in untargeted metabolomics in *Slc25a26^{KO}* hearts compared with Ctrl at P14 (n=7 for Ctrl, n=5 for *Slc25a26^{KO}*). (**B**) Log$_2$FC of pyrimidine derivatives in untargeted metabolomics in *Slc25a26^{KO}* hearts compared with Ctrl at P14 (n=7 for Ctrl, n=5 for *Slc25a26^{KO}*). (**C**) Heat map of relative z-scores from heart proteomics data, showing proteins in the Purine metabolism KEGG pathway, *Slc25a26^{KO}* hearts compared with Ctrl at P14 (n=5 for Ctrl, n=4 for

Slc25a26^{KO}). (**D**) Heat map of relative z-scores from heart proteomics data, showing proteins in the Pyrimidine metabolism KEGG pathway, *Slc25a26^{KO}* hearts compared with Ctrl at P14 (n=5 for Ctrl, n=4 for *Slc25a26^{KO}*). Data in (A and B) are presented as $\log_2$FC ± s.e.m.; statistical significance was determined using a two-tailed unpaired Student's t-test; ns not significant, *p< 0.05, **p< 0.01, ***p< 0.001. Significance analysis for (C and D) was performed using a linear model with moderated t-statistics. P-values were adjusted for multiple testing (Benjamini-Hochberg).

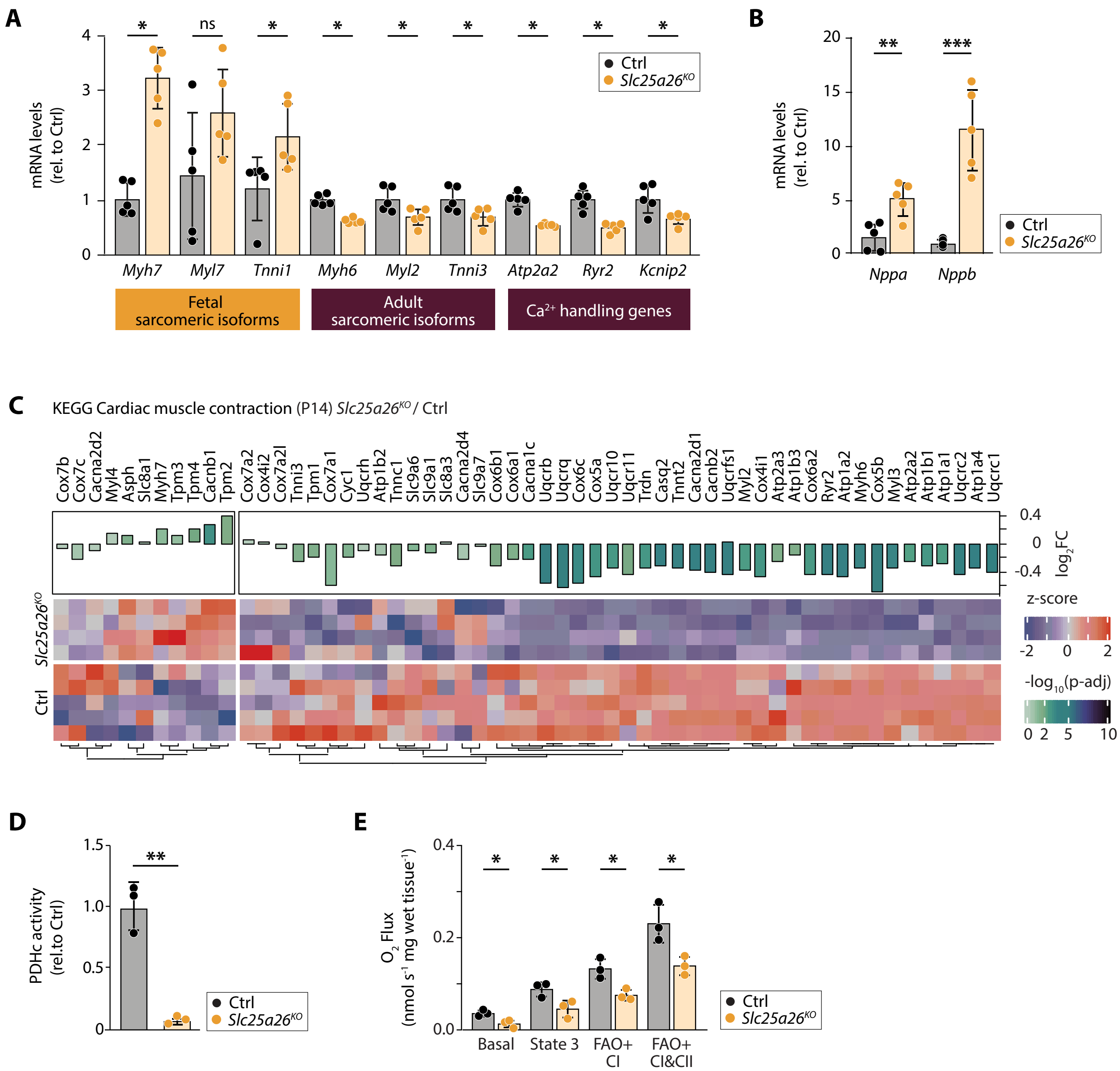

Fig. S7. In *Slc25a26^{KO}* hearts, expression of adult sarcomeric isoforms and Ca^{2+} handling genes is reduced, whereas fetal isoforms and hypertrophic genes are elevated.

(**A**) Transcript levels of the indicated Ca^{2+} handling genes and of fetal and adult sarcomeric gene isoforms in Ctrl and *Slc25a26^{KO}* hearts at P14, qRT-PCR (n=5 per genotype). (**B**) Transcript levels of hypertrophy markers *Nppa* and *Nppb* in Ctrl and *Slc25a26^{KO}* hearts at P14, qRT-PCR (n=5 per genotype). (**C**) Heat map of relative z-scores from heart proteomics data, showing proteins in the Cardiac muscle contraction KEGG pathway, *Slc25a26^{KO}* hearts compared with Ctrl at P14 (n=5 for Ctrl, n=4 for *Slc25a26^{KO}*). (**D**) Pyruvate dehydrogenase activity in Ctrl and *Slc25a26^{KO}* hearts at P14 (n=3 per genotype). (**E**) Mitochondrial respiration in permeabilized left ventricular

myocardium, using palmitoyl-carnitine as a substrate (n=3 per genotype). Data in (A, B, D, E) are presented as mean ± SD; each dot represents an individual mouse; two-tailed unpaired Student's t-test; ns = not significant, *$p < 0.05$, **$p < 0.01$, ***$p < 0.001$. Statistical analysis for (C) was performed using a linear model with moderated t-statistics. P-values were adjusted for multiple testing (Benjamini-Hochberg).

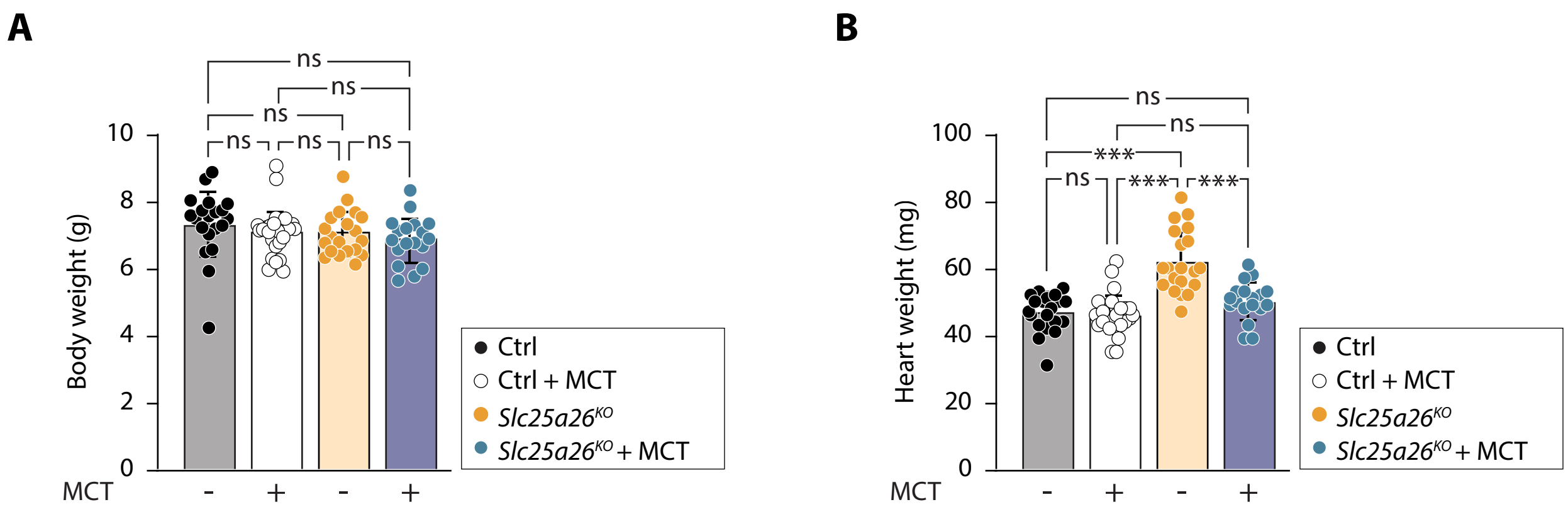

Fig. S8. Dietary supplementation with medium-chain triglycerides (MCT) restores cardiac mass.

(**A**) Body weight of control (Ctrl) and *Slc25a26*^{KO} animals fed normal or MCT-rich chow at P14 (n=21, 26, 20, 18 for Ctrl, Ctrl+MCT, *Slc25a26*^{KO}, and *Slc25a26*^{KO}+MCT, respectively). (**B**) Heart weight of control (Ctrl) and *Slc25a26*^{KO} animals fed normal or MCT-rich chow at P14 (n=21, 26, 20, 18 for Ctrl, Ctrl+MCT, *Slc25a26*^{KO}, and *Slc25a26*^{KO}+MCT, respectively).

Data are presented as mean ± SD, each dot represents an individual mouse, two-way ANOVA with Tukey's multiple comparisons test; ns=not significant, ***p< 0.001.

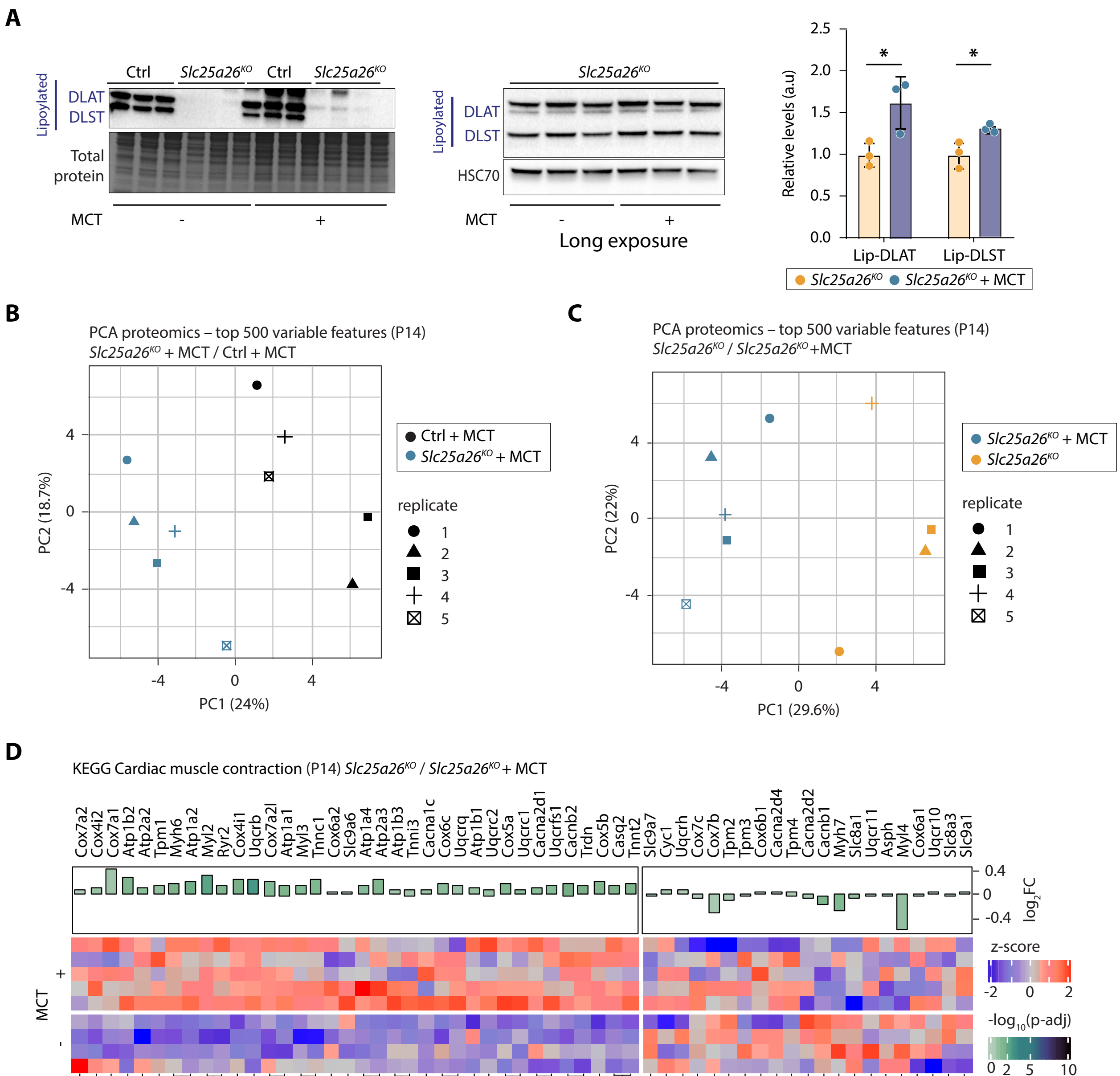

Fig. S9. Dietary supplementation with medium-chain triglycerides (MCT) improves cardiac health in *Slc25a26^{KO}* mice.

(**A**) Immunoblot of Ctrl and *Slc25a26^{KO}* heart lysates fed normal or MCT-rich chow at P14, depicting lipoylated forms of DLAT and DLST; HSC70 was used as the loading control (n=3 per genotype). On the right, quantification of a long-exposure immunoblot of *Slc25a26^{KO}* heart lysates fed normal or MCT-rich chow at P14. (**B**) PCA of LC-MS/MS label-free whole-heart proteomics, Ctrl and *Slc25a26^{KO}* fed MCT-rich chow at P14 (n=5 per genotype). (**C**) PCA of LC-MS/MS label-free whole-heart proteomics, *Slc25a26^{KO}* fed normal or MCT-rich chow at P14 (n=5 per genotype). (**D**) Heat map of relative z-scores from heart proteomics data, showing proteins in the KEGG

cardiac muscle contraction pathway, *Slc25a26^{KO}* hearts fed normal (n=4) versus MCT-rich (n=5) chow at P14. Statistical analysis was performed using a linear model with moderated t-statistics. P-values were adjusted for multiple testing (Benjamini-Hochberg). Data in (D) are presented as mean ± SD; each dot represents an individual mouse; two-tailed unpaired Student's t-test, $*p<0.05$.

Table S1.

**Probe**	**Source**	**Catalog number**
Slc25a26 (Mm00470957_m1)	ThermoFisher	Cat#4351372
Nppa (Mm01255747_g1)	ThermoFisher	Cat#4331182
Nppb Mm01255770_g1	ThermoFisher	Cat#4331182
Myh7 Mm00600555_m1	ThermoFisher	Cat#4331182
Myh6 Mm00440359_m1	ThermoFisher	Cat#4331182
Myl7 (Mm00491655_m1)	ThermoFisher	Cat#4331182
Myl2 (Mm00440384_m1)	ThermoFisher	Cat#4331182
Tnni1 (Mm00502426_m1)	ThermoFisher	Cat#4331182
Tnni3 (Mm00437164_m1)	ThermoFisher	Cat#4331182
Ryr2 (Mm00465877_m1)	ThermoFisher	Cat#4331182
Atp2a2 (Mm01201431_m1)	ThermoFisher	Cat#4331182
Kcnip2 (Mm00518915_g1)	ThermoFisher	Cat#4331182
Actb (Mm01205647_g1)	ThermoFisher	Cat#4331182
18S (Mm03928990_g1)	ThermoFisher	Cat#4331182
Hprt (Mm03024075_m1)	ThermoFisher	Cat#4331182
Nd1 (Mm04225274_s1)	ThermoFisher	Cat#4351370
Nd2 (Mm04225288_s1)	ThermoFisher	Cat#4351370
Nd3 (Mm04225292_g1)	ThermoFisher	Cat#4351370
Nd4L/Nd4 (Mm04225294_s1)	ThermoFisher	Cat#4351370
Nd5 (AIHSNT9)	ThermoFisher	custom made
Nd6 (AIVI3E8)	ThermoFisher	custom made
Cytb (Mm04225271_g1)	ThermoFisher	Cat#4351370
Cox1 (Mm04225243_g1)	ThermoFisher	Cat#4351370
Cox2 (Mm03294838_g1)	ThermoFisher	Cat#4351370
Cox3 (Mm04225261_g1)	ThermoFisher	Cat#4351370
Atp6 (Mm03649417_g1)	ThermoFisher	Cat#4351370
Atp8 (Mm04225236_g1)	ThermoFisher	Cat#4351370
mtRnr1 (Mm04260177_s1)	ThermoFisher	Cat#4351370
mtRnr2 (Mm04260181_s1)	ThermoFisher	Cat#4351370

List of TaqMan probes used in this study.

Data S1. (separate file)

Untargeted proteomics data set of P7 KO vs Ctrl

Untargeted proteomics data set of P14 NCD KO vs NCD Ctrl

Untargeted proteomics data set of P14 MCT KO vs MCT Ctrl

Untargeted proteomics data set of P14 MCT KO vs NCD KO

Data S2. (separate file)

Untargeted metabolomics data set of P7 KO vs Ctrl

Untargeted metabolomics data set of P14 NCD KO vs NCD Ctrl

Untargeted metabolomics data set of P14 MTC KO vs NCD KO

Data S3. (separate file)

Redox proteome data set

Data S4. (next page)

Images of uncropped Western blots

Fig.1D

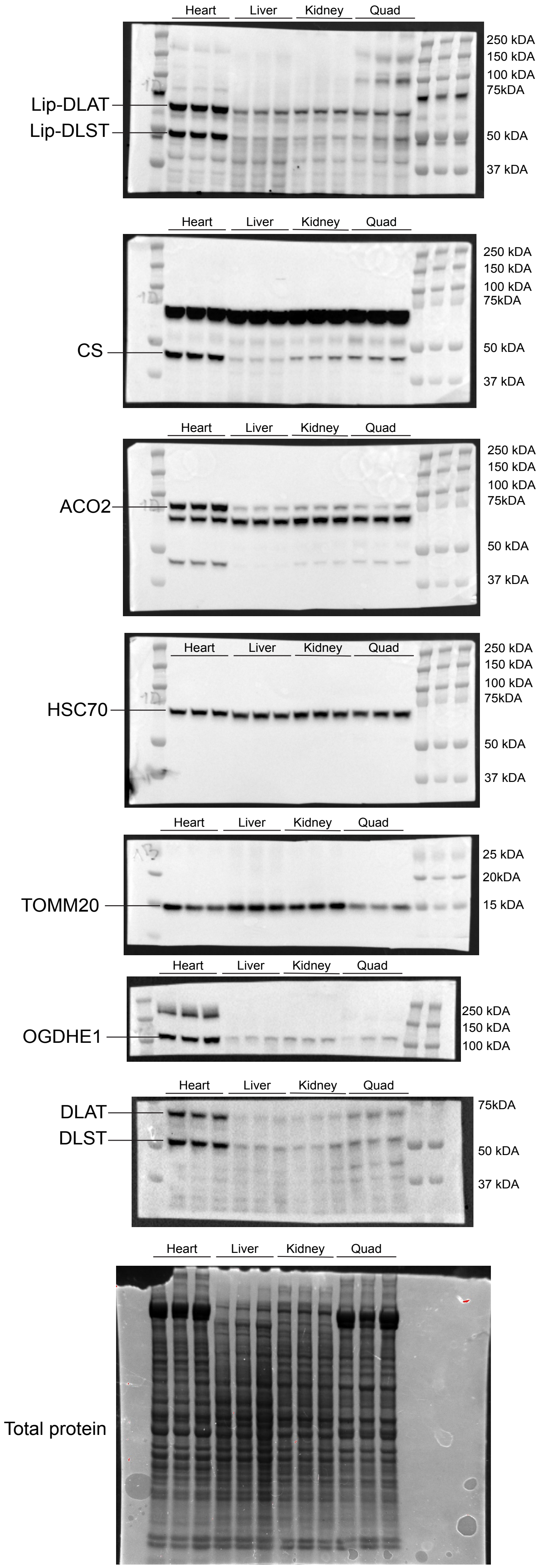

Heart
Liver
Kidney
Quad
Lip-DLAT
Lip-DLST
250 kDA
150 kDA
100 kDA
75kDA
50 kDA
37 kDA
CS
ACO2
HSC70
TOMM20
25 kDA
20kDA
15 kDA
OGDHE1
DLAT
DLST
Total protein

Fig.1E

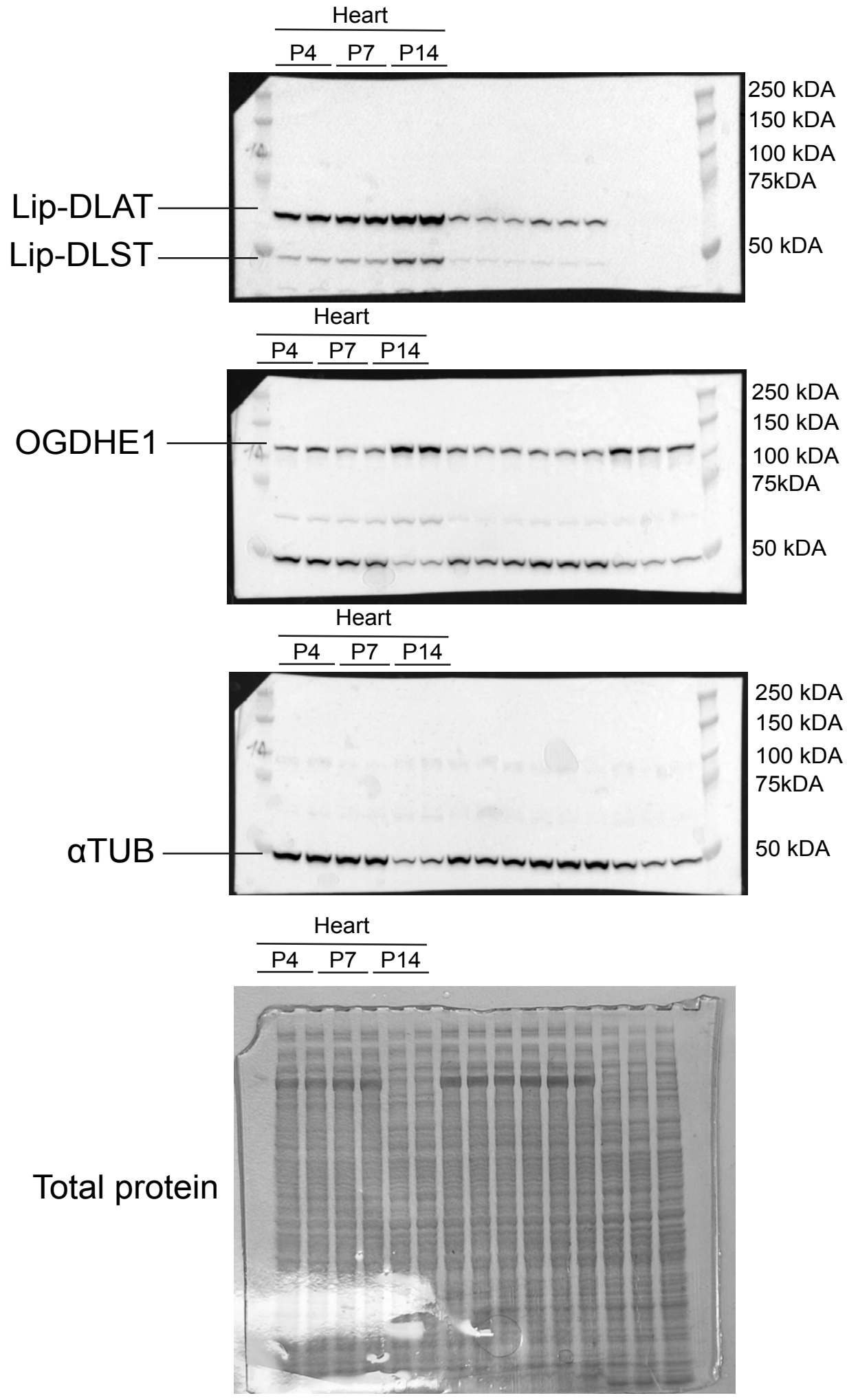
Heart
P4
P7
P14
250 kDA
150 kDA
100 kDA
75kDA
50 kDA
Lip-DLAT
Lip-DLST
Heart
P4
P7
P14
250 kDA
150 kDA
100 kDA
75kDA
50 kDA
OGDHE1
Heart
P4
P7
P14
250 kDA
150 kDA
100 kDA
75kDA
50 kDA
αTUB
Heart
P4
P7
P14
Total protein

Fig.2H
P0-1

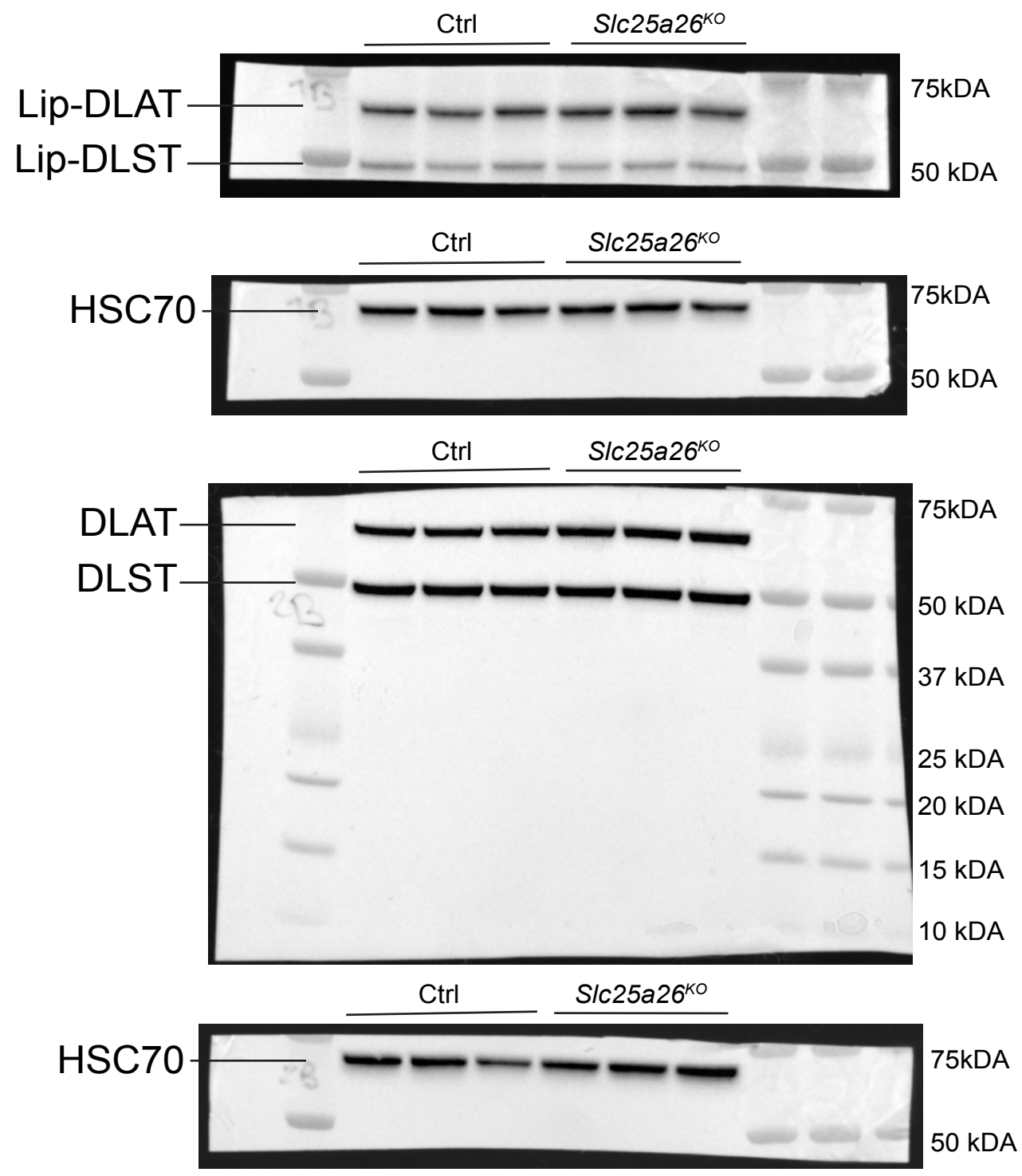

Fig.2E
P7

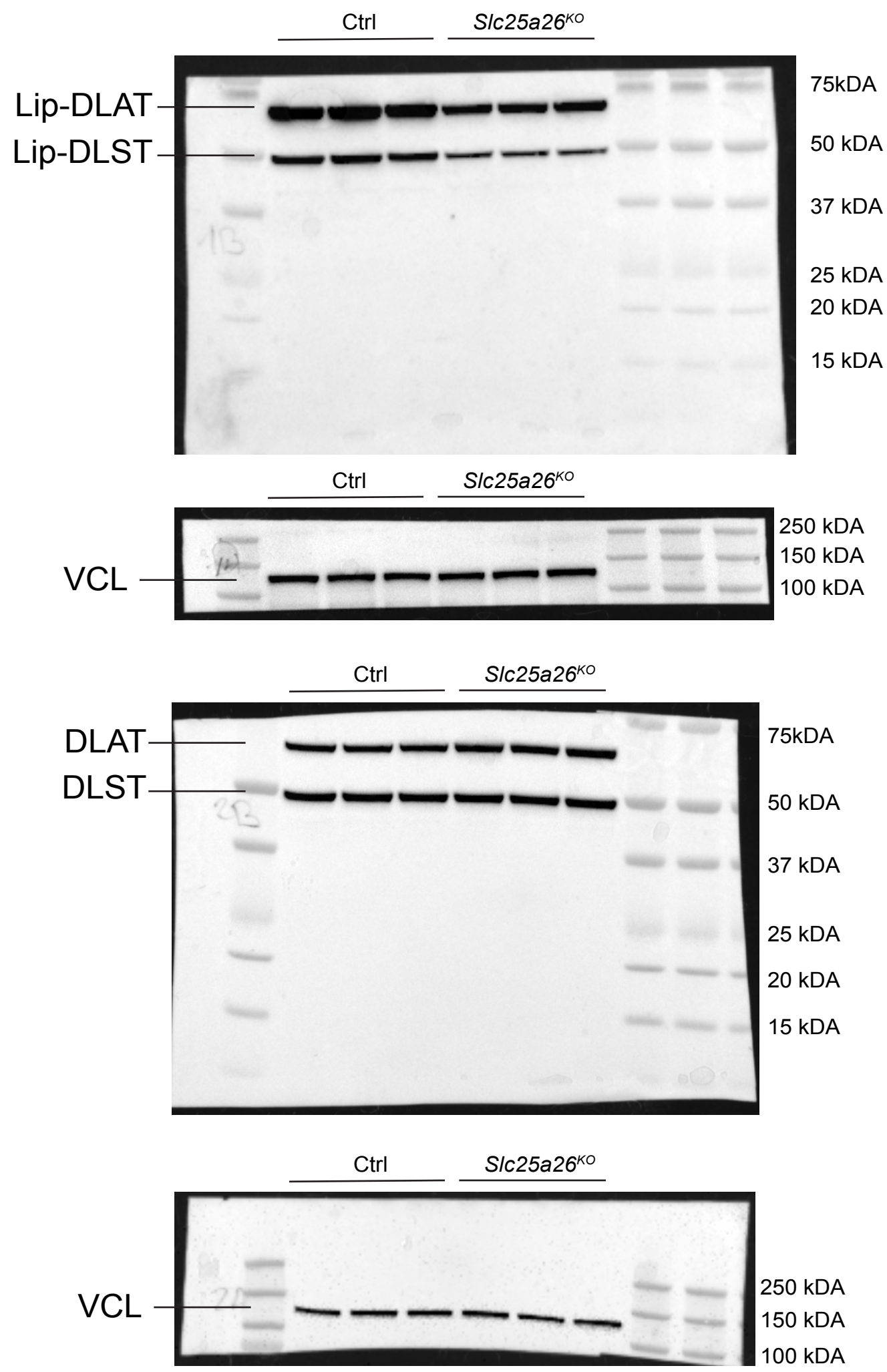

Fig.4E
Heart P14

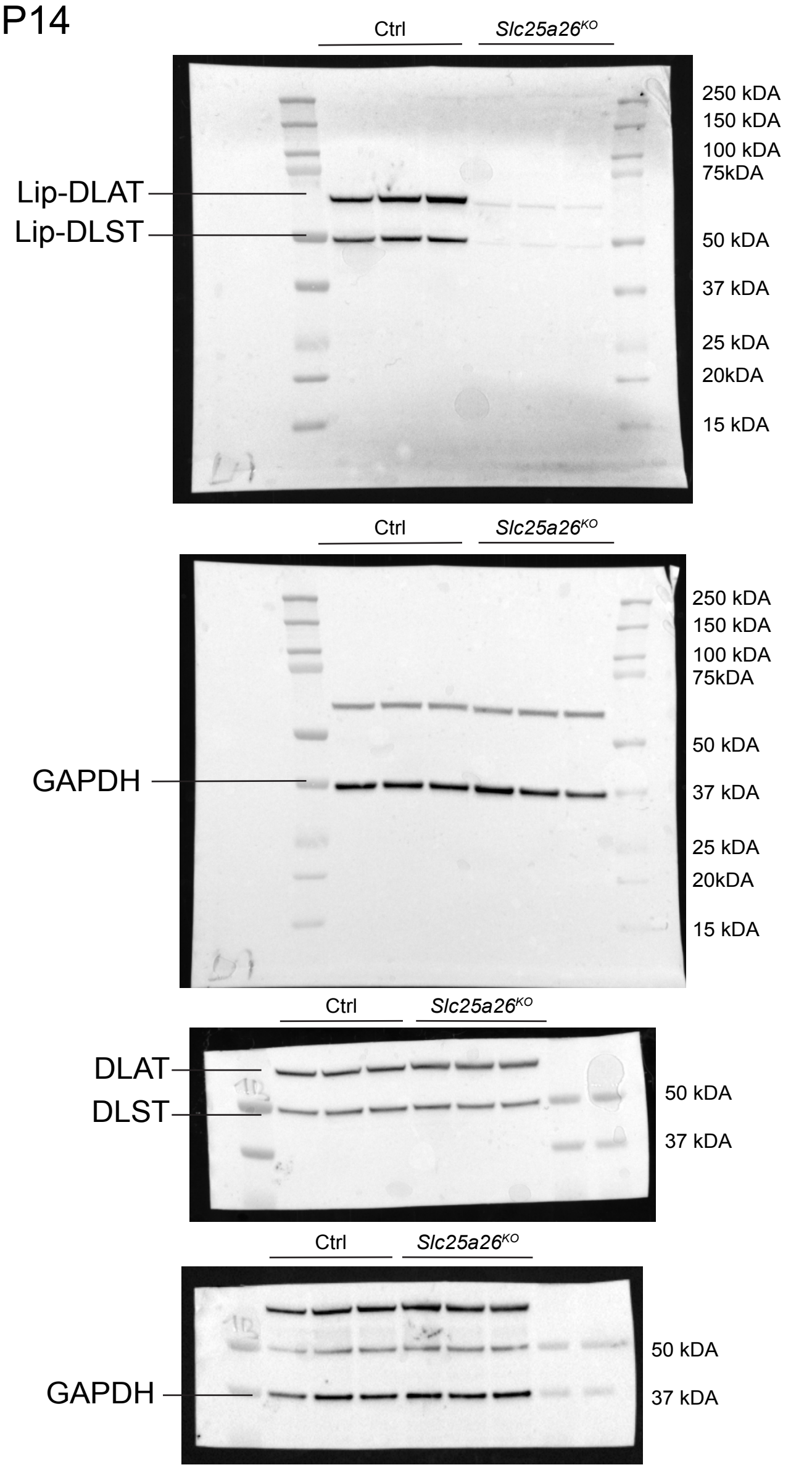

Fig.4F
Quadriceps P14

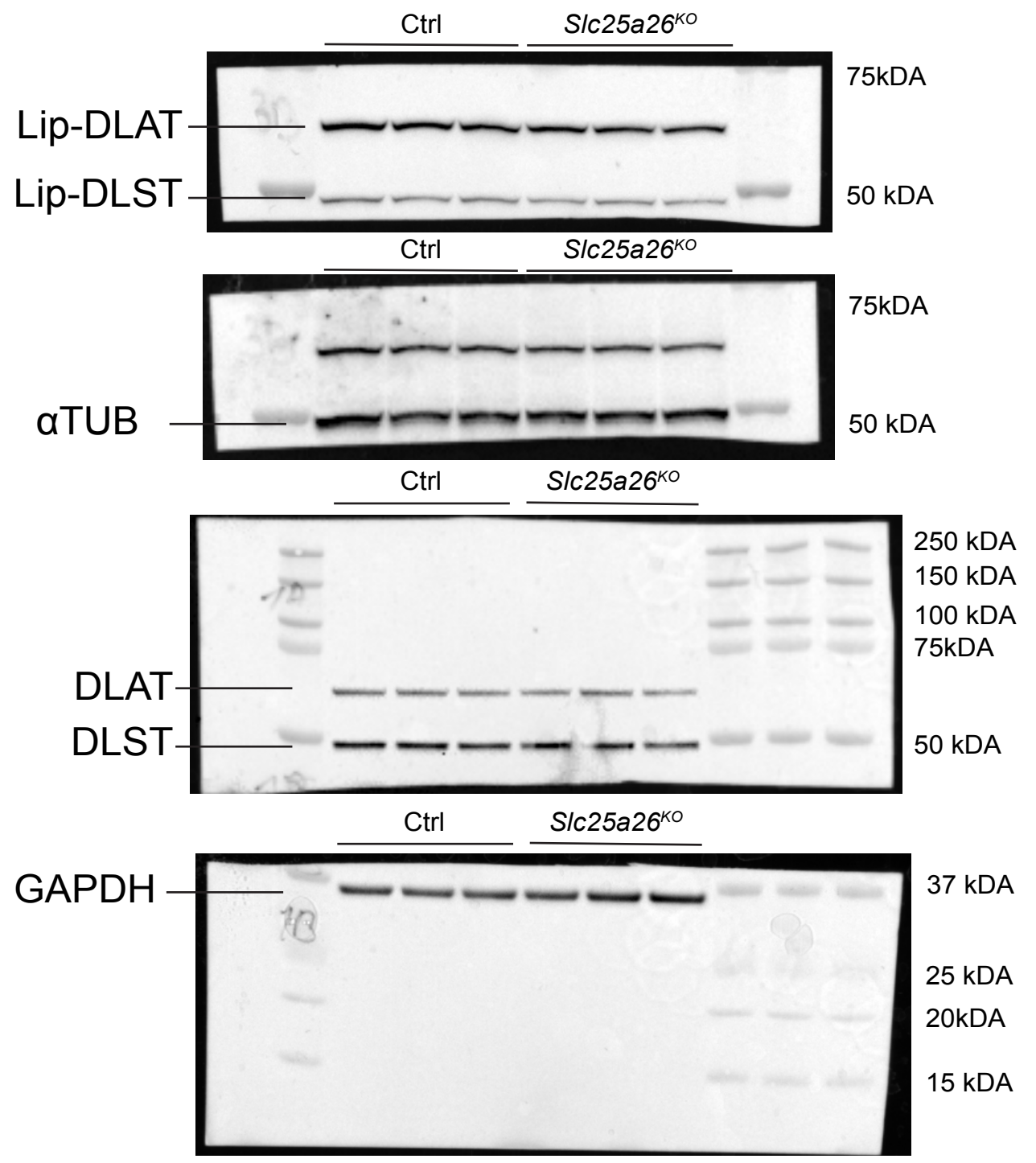

Fig.5I

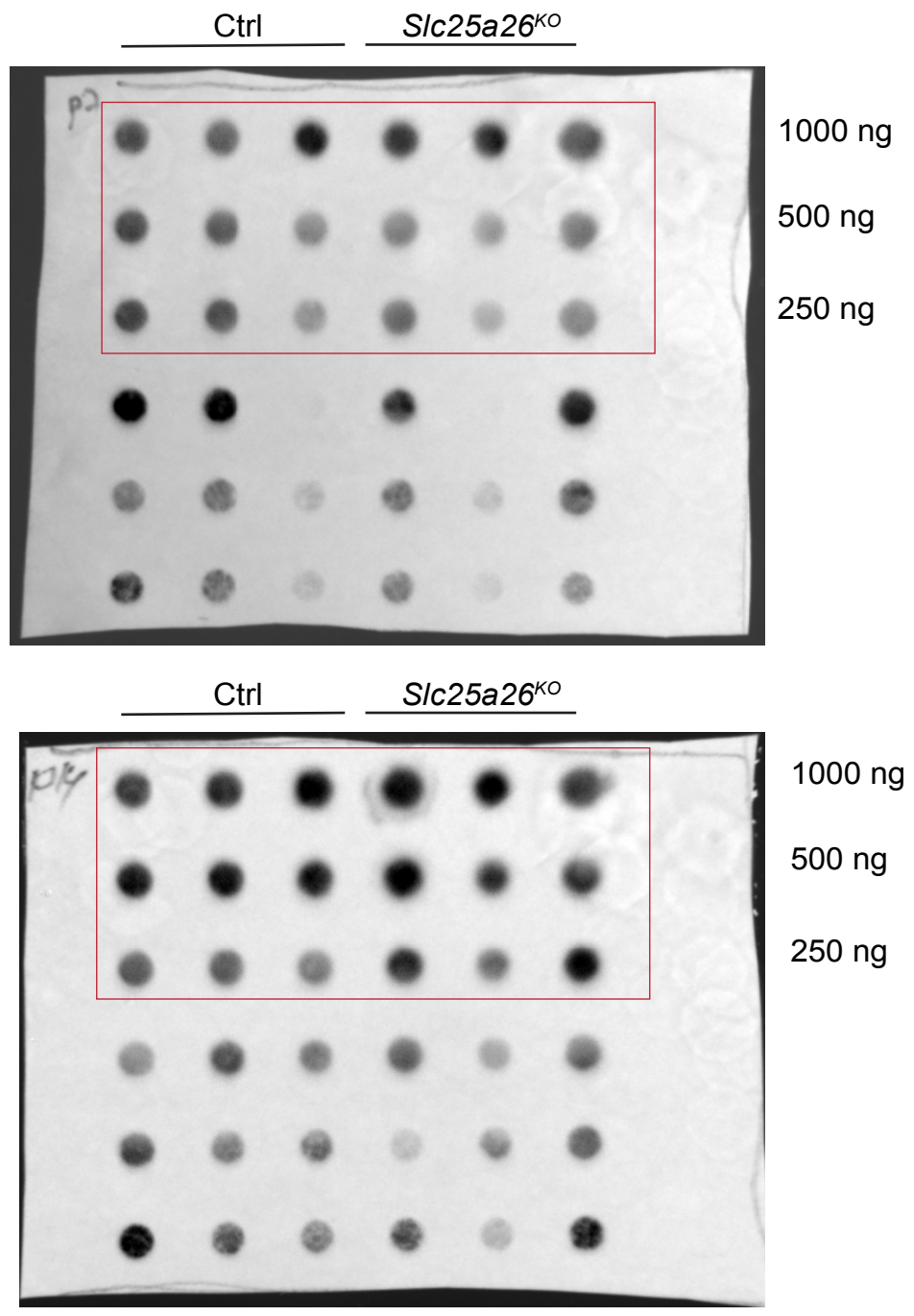
Ctrl
Slc25a26^KO
1000 ng
500 ng
250 ng
Ctrl
Slc25a26^KO
1000 ng
500 ng
250 ng

Fig.6B

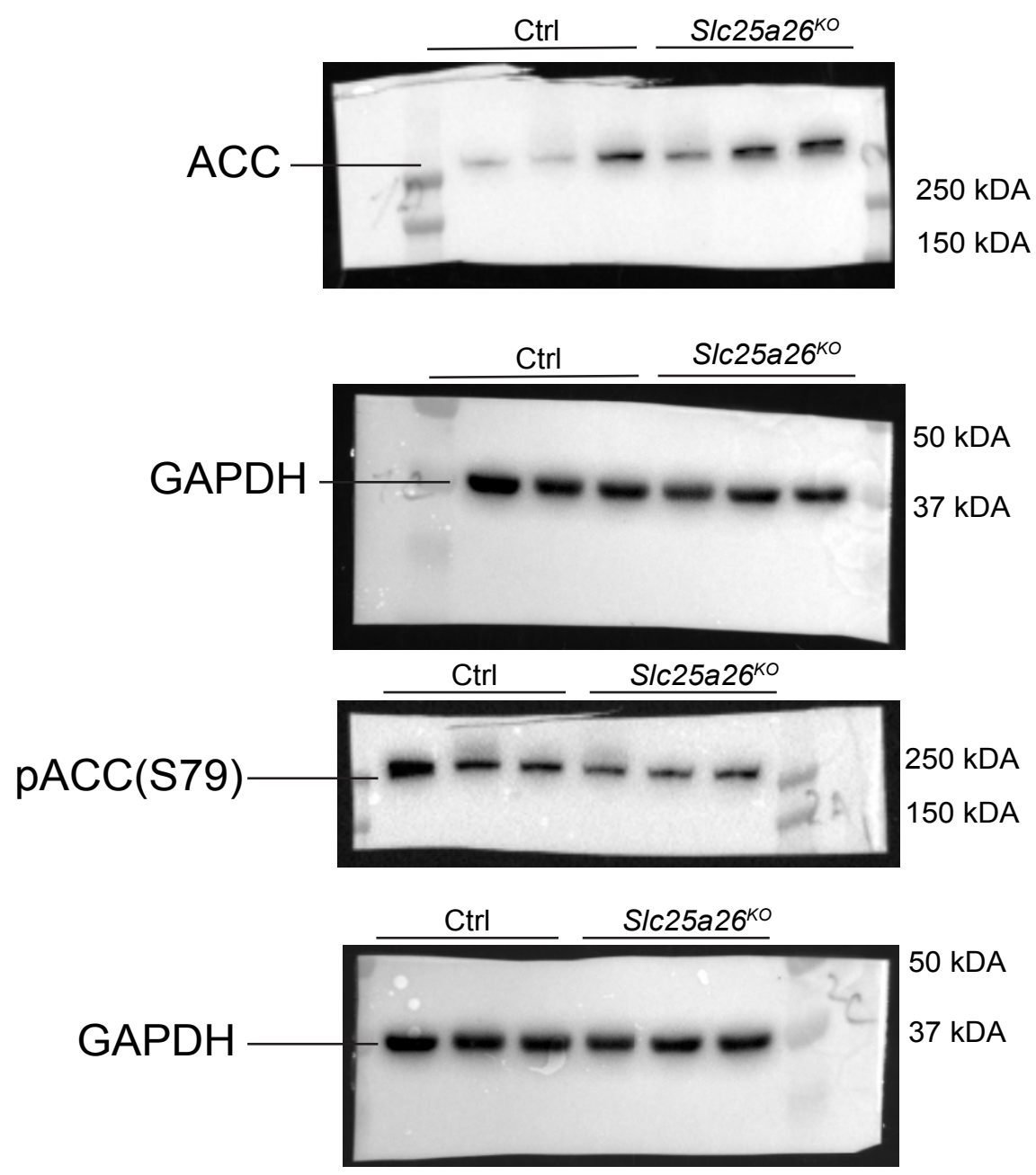
Ctrl
Slc25a26^KO
ACC
250 kDA
150 kDA
Ctrl
Slc25a26^KO
GAPDH
50 kDA
37 kDA
Ctrl
Slc25a26^KO
pACC(S79)
250 kDA
150 kDA
Ctrl
Slc25a26^KO
GAPDH
50 kDA
37 kDA

Fig.S2E

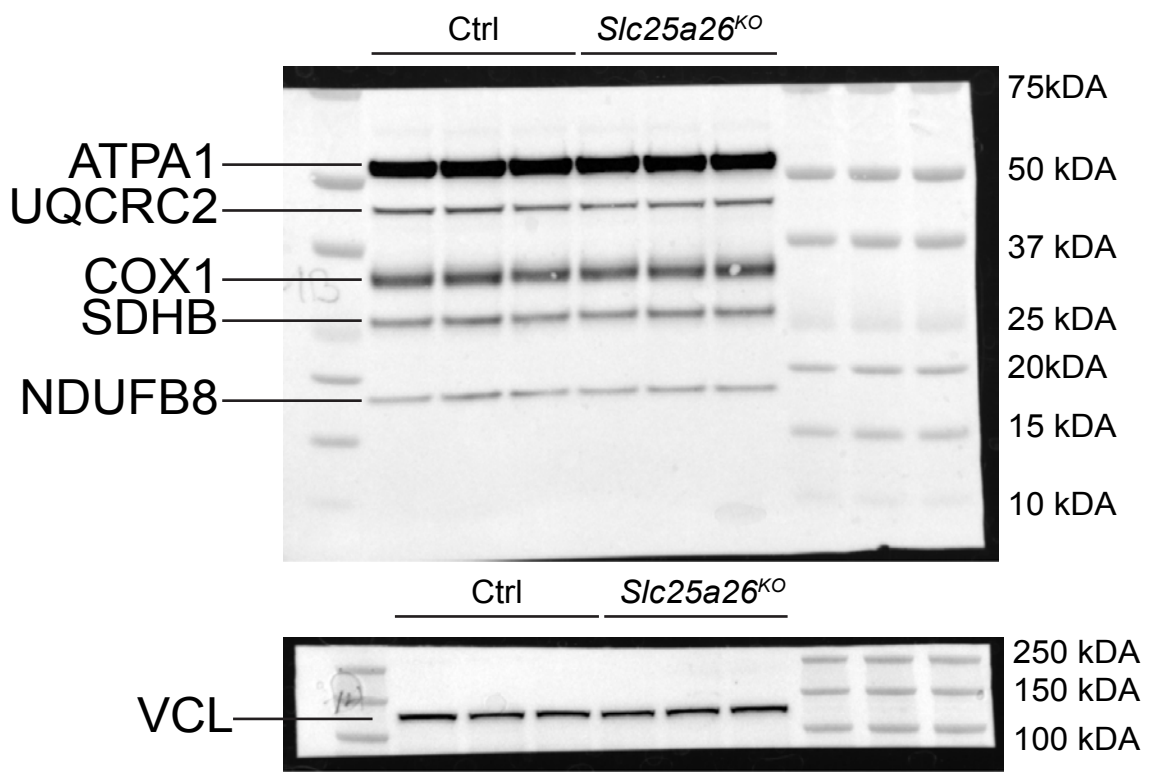

Fig.S3D

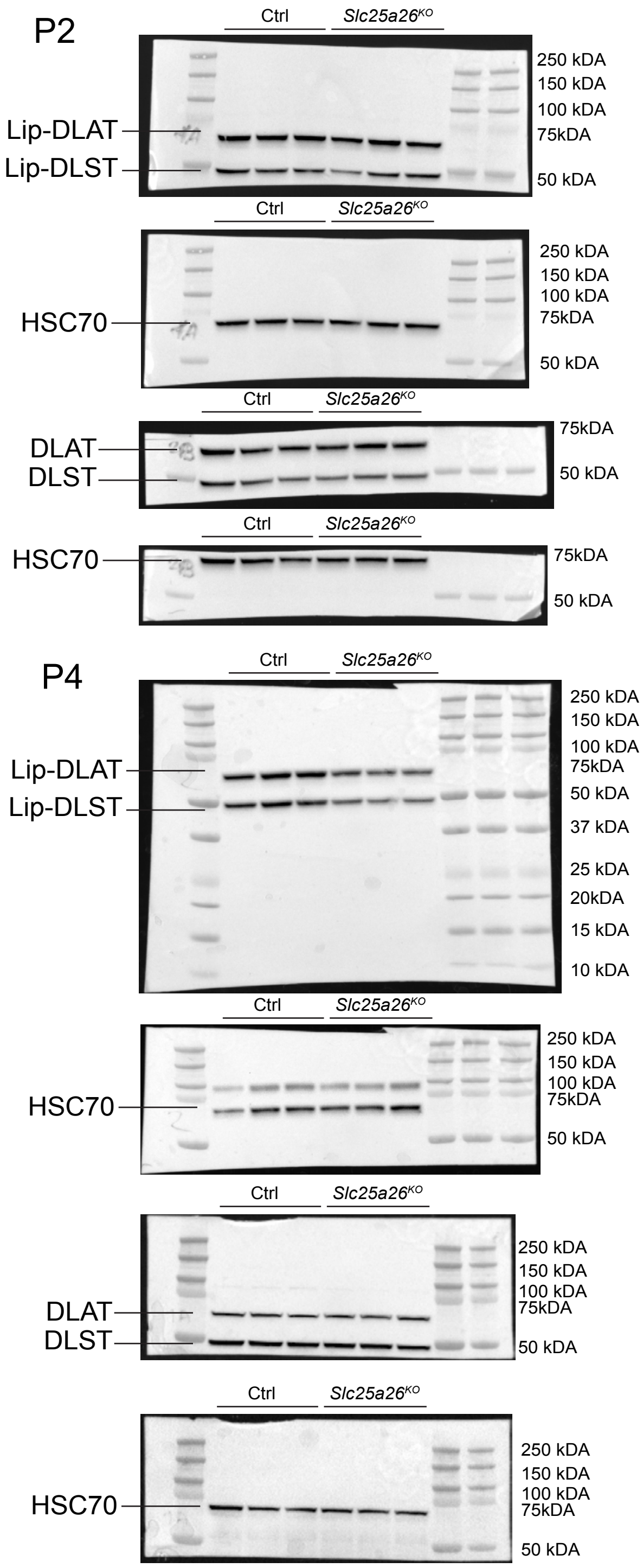

Fig.S5B

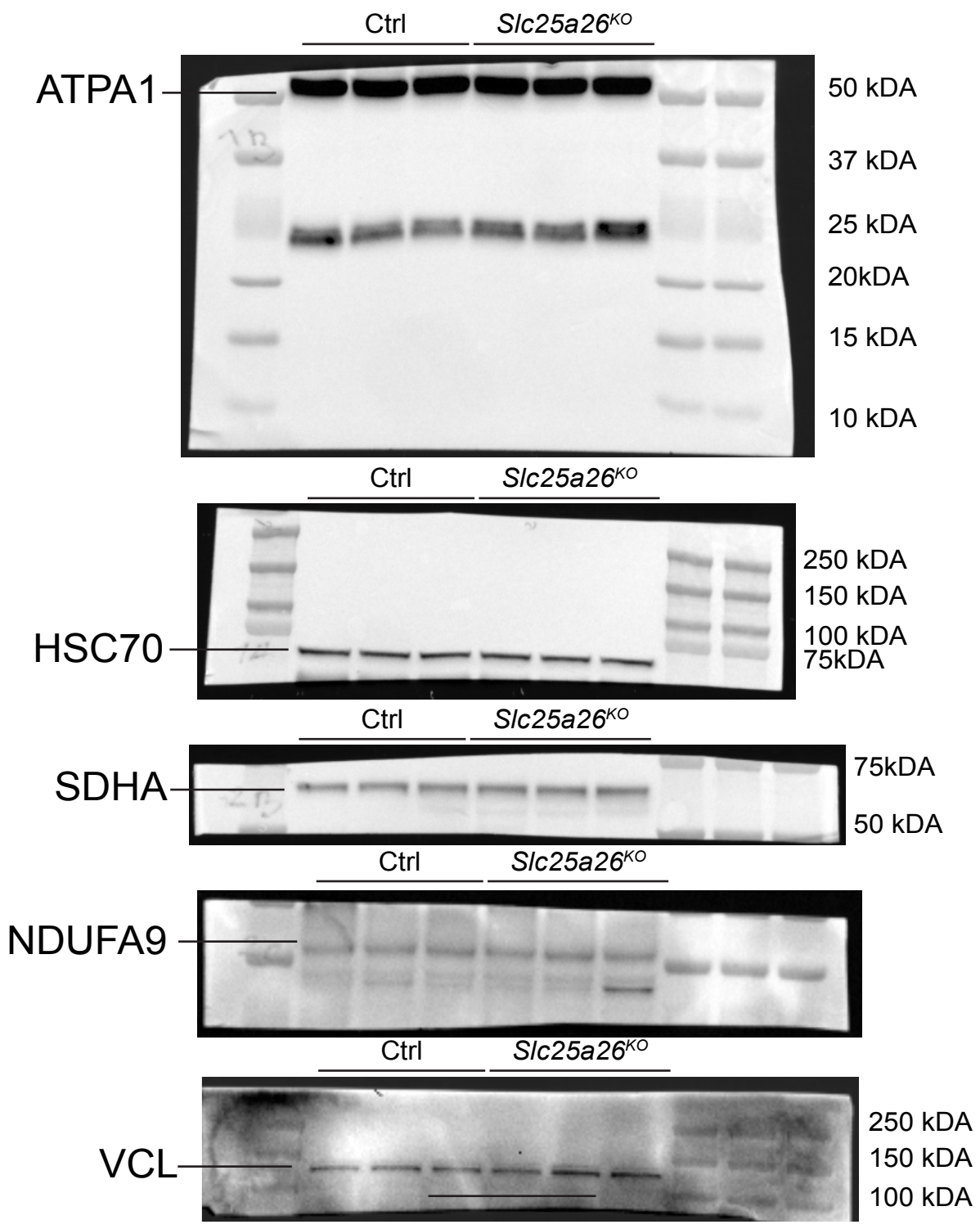

Fig.S9A

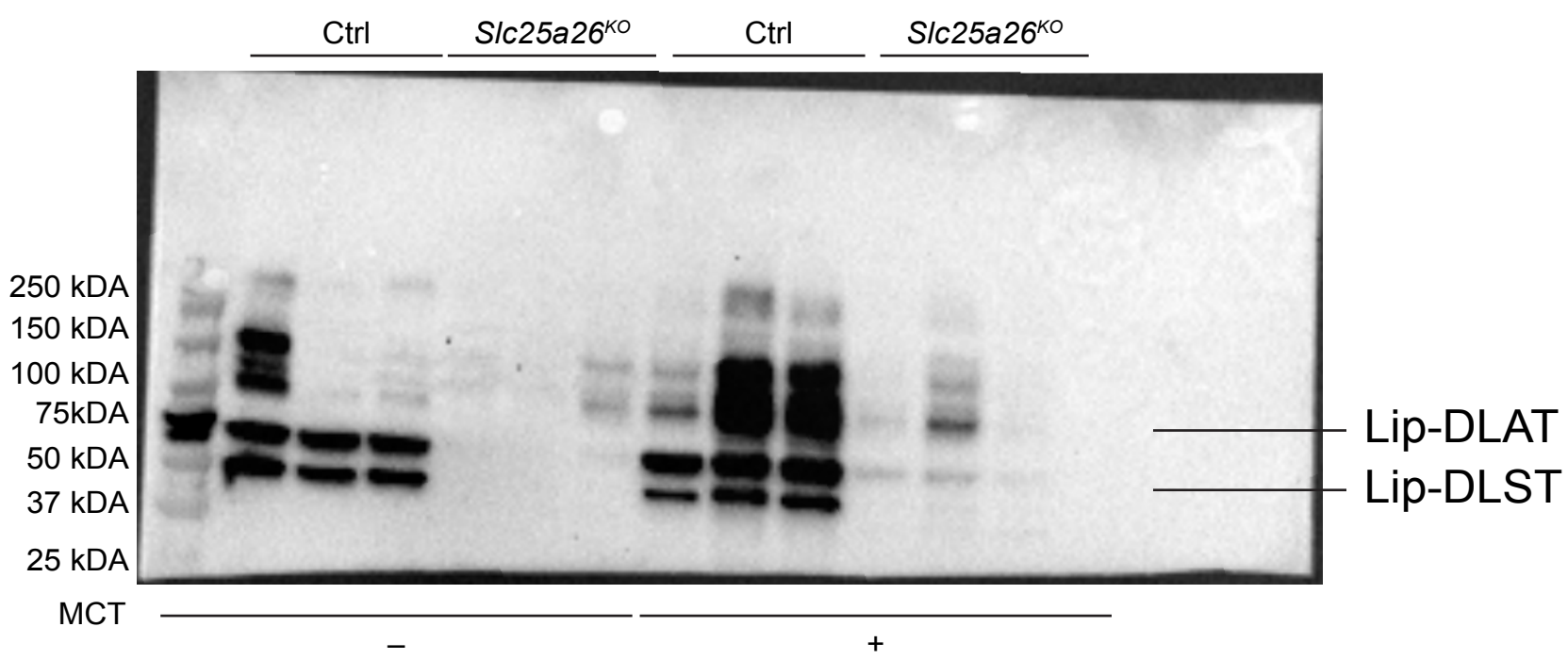

Supplement: Supplementary file 1 [file sciadv.aeg8792_sm.pdf]
